# Targeting the Mapk13‐Tcf1‐Slc7a5 Axis via One‐Carbon Metabolic Regulation to Prevent Chronic Allograft Vasculopathy

**DOI:** 10.1002/advs.202520815

**Published:** 2026-01-15

**Authors:** Wang Yi, Di Wu, Jing Liu, Shi Chen, Liu Song, Bin Xie, Aini Xie, Peixiang Lan, Zhishui Chen

**Affiliations:** ^1^ Institute of Organ Transplantation Tongji Hospital Tongji Medical College Huazhong University of Science and Technology Wuhan Hubei China; ^2^ Key Laboratory of Organ Transplantation Ministry of Education Chinese Academy of Medical Sciences Wuhan Hubei China; ^3^ NHC Key Laboratory of Organ Transplantation Wuhan Hubei China; ^4^ Pancreas Center First Affiliated Hospital of Nanjing Medical University Nanjing China; ^5^ State Key Laboratory for Diagnosis and Treatment of Severe Zoonotic Infectious Diseases Huazhong University of Science and Technology Wuhan Hubei China

**Keywords:** chronic allograft vasculopathy, methionine restriction, one‐carbon metabolism, stem cell‐like T cells, TCF1

## Abstract

Chronic allograft vasculopathy (CAV) is driven in part by stem‐like CD4^+^ T cells, but how these cells sustain their progenitor programs during chronic rejection remains unclear. Here, a metabolic‐epigenetic axis is identified in which Mapk13 phosphorylates Tcf1 at T289, enabling Tcf1 to activate the amino acid transporter Slc7a5 and enhance methionine uptake. This rewires one‐carbon metabolism and increases H3K4me3 enrichment at the *Tcf7* locus, thereby maintaining stem‐like CD4^+^ T cells within rejecting grafts. Disruption of this circuit‐via genetic deletion of Mapk13 or Slc7a5, or through dietary methionine restriction‐reduces Tcf1^+^ CD4^+^ T cell stemness and prevents CAV in mouse models. These findings reveal the Mapk13‐Tcf1‐Slc7a5 axis as a critical metabolic dependency of pathogenic T cells and highlight one‐carbon metabolism as a promising target to promote long‐term graft survival.

## Graphical abstract

This study emphasizes the role of the Mapk13‐Tcf1‐Slc7a5‐methionine metabolism axis in stem‐like CD4^+^ T cells. Moreover, it uncovers the mechanism through which limiting one‐carbon metabolism in CD4^+^ stem‐like T cells suppresses the tide of chronic allograft vasculopathy, offering potential targets to promote long‐term graft survival.

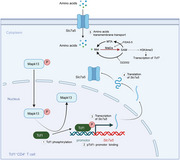

## 1 Introduction

Chronic allograft vasculopathy (CAV) represents a form of immune‐mediated allograft injury that occurs in transplanted kidneys during chronic rejection. CAV represents a localized manifestation of chronic rejection that affects the arteries of transplanted kidneys and serves as a hallmark histopathological feature of chronic rejection in all vascularized transplants. Its pathological characteristics include arterial hyperplasia and thickening in various branches of the graft's arteries, leading to luminal narrowing or complete occlusion. This process leads to ischemic atrophy of the graft parenchyma and subsequent fibrous tissue hyperplasia, ultimately culminating in widespread fibrosis and necrosis [1].

CD4^+^ T cells play a pivotal role in the development of CAV. Upon encountering an antigen, naive CD4^+^ T cells activate and differentiate into various effector helper T cell subtypes, contributing to vascular injury in transplanted allografts [2, 3]. Multiple studies demonstrate that CD4^+^ effector T cells alone are sufficient to drive chronic rejection and transplant vasculopathy, as their selective presence consistently induces intimal hyperplasia and progressive vascular injury across cardiac allograft models [4, 5, 6, 7]. Although the primary events and molecular pathways that trigger T cell activation are well‐characterized, the mechanisms that maintain CD4^+^ T cell stem cell‐like traits remain poorly understood [8]. The metabolic profiles of antigen‐induced stem cell‐like CD4^+^ T cells represent a key area of recent progress. These findings underscore the stem cell‐like traits of Tcf1^+^CD4^+^ T cells, characterized by self‐renewal and subsequent effector differentiation potential. Additional studies are required to identify the intrinsic mechanisms that control self‐renewal in CD4^+^ T cells and determine to what extent they shape CD4^+^ T cell responses.

Certain transcription factors, including Tcf1, Klf2, and Lef1, are known to be pivotal in promoting or maintaining T cell stemness [9]. Our previous study reported that Tcf1 in tumor‐reactive CD8^+^ T cells is regulated by Mapk13 phosphorylation [10]. Within CD4^+^ T cell subsets, Tcf1 phosphorylation is less well understood. It remains unclear how the stem cell‐like characteristics of CD4^+^ T cells are regulated at the molecular level. Metabolic pathways are controlled by members of the solute carrier family, who are instrumental in orchestrating the intake and efflux of a broad spectrum of metabolites, such as lipids, amino acids, and ions. Growing evidence suggests that metabolic pathways dictate T cell fate and shape their epigenetic and functional profiles [11]. Methionine acts as a universal methyl donor for histone methylation and is crucial in driving epigenetic reprogramming in CD4^+^ helper T cells.

Here, we demonstrate that Mapk13 phosphorylates Tcf1 in CD4^+^ T cells to initiate a stem‐like transcriptional program. Tcf1 subsequently upregulates Slc7a5 expression, thereby facilitating increased methionine uptake. Intracellular methionine serves as a methyl donor for the methionine cycle and modulates H3K4me3 deposition at the Tcf7 locus, which enhances Tcf7 transcription and sustains the stemness of CD4^+^ T cells. Accordingly, blockade of the Mapk13‐Tcf1‐Slc7a5‐methionine metabolic pathway disrupts CD4^+^ T cell stemness and ultimately alleviates CAV. Collectively, our findings uncover a surprising mechanism whereby coordinated metabolic and epigenetic remodeling in CD4^+^ T cells promotes graft survival.

## 2 Results

### 2.1 TCF1^+^CD4^+^ T Cells Accumulate in Transplanted Kidneys in Chronic T Cell‐Mediated Rejection

**FIGURE 1:**
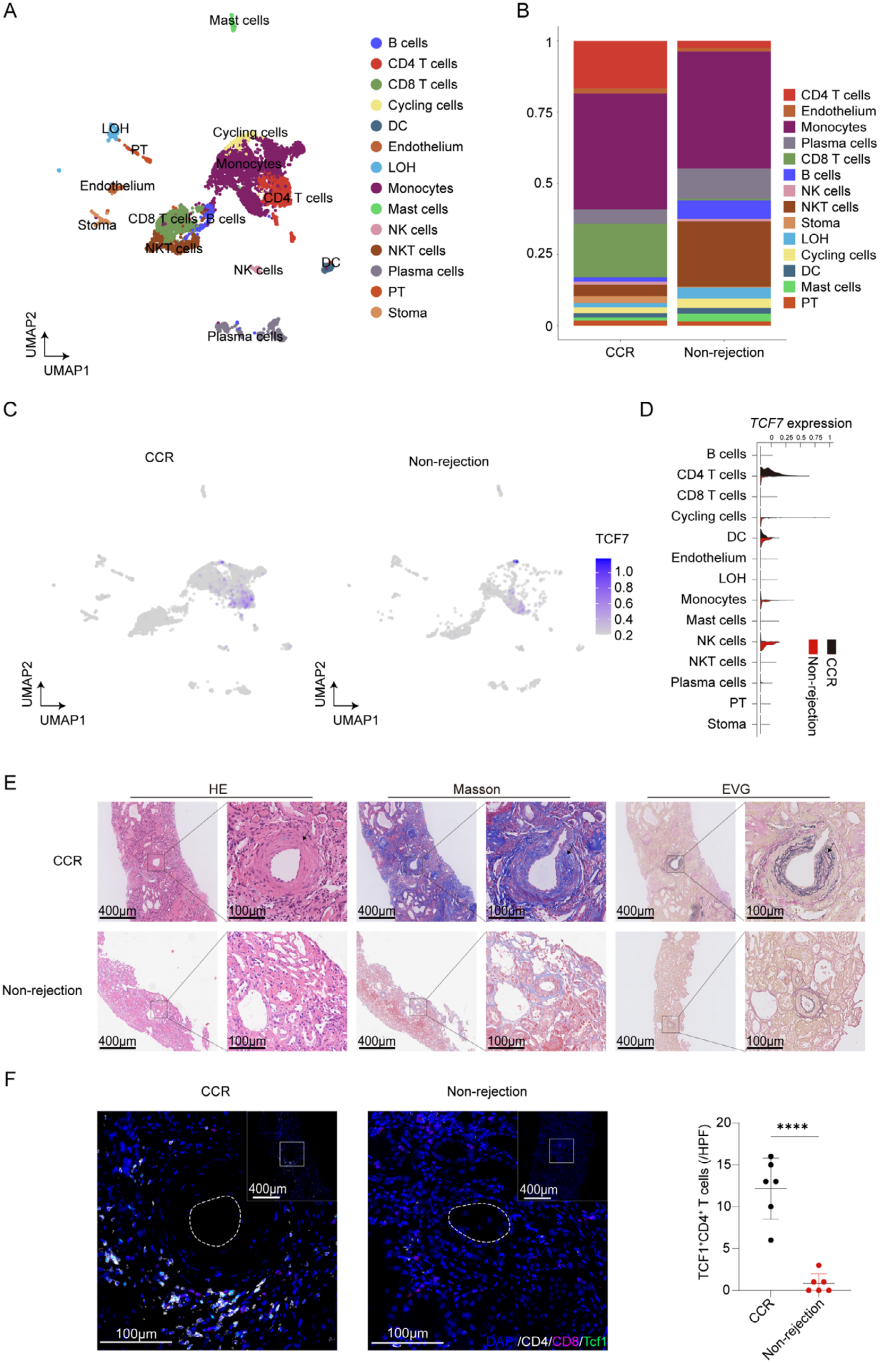
TCF1 expression profile in kidney transplant with chronic cellular rejection versus non‐rejection. (A) UMAP plots illustrating the major immune and parenchymal cell populations identified by single‐cell RNA sequencing in human renal allograft biopsies. (B) Relative proportions of the major immune and parenchymal cell populations in allografts diagnosed with chronic cellular rejection versus non‐rejection. (C) UMAP visualization of TCF7 expression across the identified cell clusters. (D) Violin plot quantifying TCF7 expression levels across different cell populations. (E) Representative histological staining of kidney allograft sections with chronic cellular rejection compared with non‐rejection allografts (*n* = 6). (F) Representative mIHC staining of kidney allograft sections with chronic cellular rejection compared with non‐rejection allografts (*n* = 6). ^****^ *p* < 0.00001.

To elucidate the role of TCF1 in chronic rejection, renal allograft biopsy specimens were obtained from kidney transplant recipients diagnosed with chronic cellular rejection (CCR), as well as from recipients without evidence of rejection. These samples were subjected to single‐cell RNA sequencing, histopathological examination, and immunofluorescence staining. Following enzymatic dissociation of the biopsy tissues into single‐cell suspensions, CD45^+^ cells were isolated by flow cytometry and subsequently processed for single‐cell sequencing. All major renal parenchymal and immune cell populations were identified based on lineage‐specific marker expression (Figure 1A; Figure S1). In allografts with chronic cellular rejection, the proportions of both CD4^+^ and CD8^+^ lymphocytes were markedly elevated compared with non‐rejecting grafts (Figure 1B). At the single‐cell level, *TCF7* transcripts were predominantly expressed in CD4^+^ T cells. Notably, the abundance of CD4^+^ T cells exhibiting high *TCF7* expression was significantly increased in grafts with chronic rejection relative to those without rejection (Figure 1C,D). Additionally, transplanted kidney biopsy samples were collected for histopathological examination. Histological staining revealed significantly increased mononuclear cell infiltration in transplanted kidneys with chronic rejection compared to those without rejection. Arterial hyperplasia and thickening led to lumen narrowing or complete occlusion, with extensive fibrous tissue hyperplasia observed in transplanted kidneys with chronic rejection compared to those without rejection (Figure 1E). Multiplex immunohistochemistry (mIHC) staining further revealed significantly increased T cell infiltration in transplanted kidneys with chronic rejection compared to those without rejection, particularly with a significant increase in TCF1^+^CD4^+^ T cells (Figure 1F).

### 2.2 Mapk13 Phosphorylates Tcf1 in CD4^+^ T Cells

**FIGURE 2:**
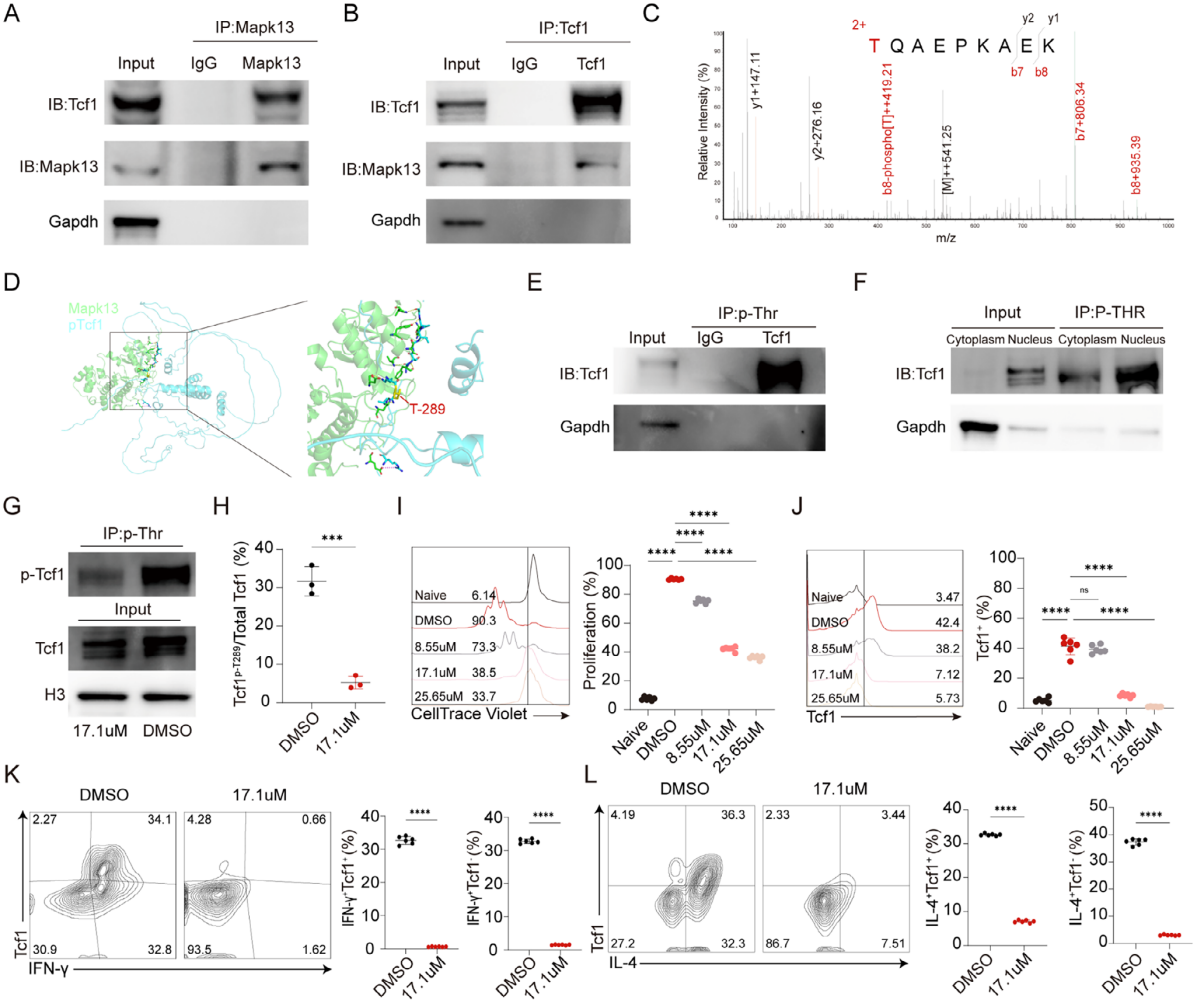
Regulation of Tcf1 phosphorylation and expression by Mapk13. (A–F) CD4^+^ T cells were isolated from the spleen and stimulated with anti‐CD3/CD28 antibodies for 72 h. The cells were then collected for co‐immunoprecipitation, mass spectrometry, IP, and immunoblots. (A,B) Co‐IP was performed to detect the interaction between Mapk13 and Tcf1. (C) Phospho‐proteomics using mass spectrometry to identify Tcf1 phosphorylation sites. (D) Interactive docking analysis predicting the binding of Mapk13 to Tcf1. (E) Immunoprecipitation was performed using anti‐Phospho‐Threonine antibodies to detect phosphorylated Tcf1. (F) Cytoplasmic and nuclear proteins were extracted separately and divided equally into two portions: one for Input and the other for IP. IP was performed to enrich phosphorylated proteins from both the cytoplasm and nucleus separately, while anti‐Tcf1 antibodies were used to detect the protein levels of total Tcf1 and phosphorylated Tcf1 in both compartments. The loading volumes for Cytoplasm Input, Nucleus Input, Cytoplasm IP, and Nucleus IP were identical. (G–J) CD4^+^ T cells were isolated from the spleen and stimulated with anti‐CD3/CD28 antibodies in the presence of MAPK13‐inhibitors or DMSO for 72 h, after which the cells were collected for IP, immunoblots, flow cytometry, and RT‐qPCR. (G) Protein levels of total Tcf1 and p‐Tcf1 in the nucleus of cells treated with MAPK13‐inhibitors compared to those treated with DMSO. (H) Relative abundance of pT289‐Tcf1 to total Tcf1 in CD4^+^ T cells treated with DMSO or MAPK13‐inhibitors (*n* = 3). (I,J) Proliferation (I) and (J) Tcf1 expression of cells treated with MAPK13‐inhibitors at varying concentrations or DMSO was assessed by flow cytometry (*n* = 6). (K–M) CD4^+^ T cells were isolated from the spleen and stimulated with anti‐CD3/CD28 antibodies in the presence of different cytokines and MAPK13‐inhibitors (17.1µm) or DMSO for 72 h, after which the cells were collected for flow cytometry analysis. Representative flow cytometry plots and proportions of cells treated with MAPK13‐inhibitors (17.1 µm) compared to those treated with DMSO (*n* = 6). (K) IL‐12 (10 ng/mL), IL‐2 (10 ng/mL), and anti‐IL‐4 (10 µg/mL) were used to induce Th1 cells. (L) IL‐4 (10 ng/mL), IL‐2 (5 ng/mL), anti‐IFN‐γ (10 µg/mL), and anti‐IL‐12 (10 µg/mL) were used to induce Th2 cells (*n* = 6). ns, not significant, ^*^ *p* < 0.01, ^**^ *p* < 0.001, ^***^ *p* < 0.0001, ^****^ *p* < 0.00001.

Our previous research demonstrated that Mapk13 acts as a kinase that phosphorylates Tcf1 in CD8^+^ T cells. However, whether Mapk13 phosphorylates Tcf1 in CD4^+^ T cells, and the specific phosphorylation sites involved, remain unknown. To validate our hypothesis, CD4^+^ T cells were isolated from the spleen and stimulated with anti‐CD3/CD28 antibodies for 72 h. We performed co‐immunoprecipitation assays using anti‐Mapk13 antibodies and anti‐Tcf1 antibodies. The co‐IP results confirmed that Mapk13 interacts with Tcf1 (Figure 2A,B). To determine whether Mapk13 phosphorylated Tcf1 and to identify the specific phosphorylation site, we performed an IP‐MS assay using anti‐Mapk13 antibodies. Mass spectrometry analysis revealed that Tcf1 was phosphorylated at threonine 289 (Figure 2C). Z‐DOCK simulated the interaction between Mapk13 and phosphorylated Tcf1 (pT289‐Tcf1), with the highest‐ranked complex scoring 1312.603 (Figure 2D). To confirm the phosphorylation of Tcf1 by Mapk13, we performed an immunoprecipitation assay using an anti‐phospho‐threonine antibody. The results demonstrated that Tcf1 is phosphorylated by Mapk13 within the nucleus (Figure 2E,F). Additionally, treatment of CD4^+^ T cells with MAPK13‐inhibitors (17.1 µm) resulted in a significant decrease in both total Tcf1 and p‐Tcf1 levels (Figure 2G; Figure S2). Immunoprecipitation followed by mass spectrometry revealed that in CD4^+^ T cells treated with MAPK13‐inhibitors, the ratio of pT289‐Tcf1 to total Tcf1 was significantly reduced compared with controls (Figure 2H). To investigate the effects of Mapk13 inhibition on CD4^+^ T cell proliferation and differentiation, activated CD4^+^ T cells were treated with varying doses of MAPK13‐inhibitors or DMSO. The results demonstrated that MAPK13‐inhibitors suppressed CD4^+^ T cell proliferation and reduced Tcf1 expression in a dose‐dependent manner (Figure 2I,J). Furthermore, MAPK13‐inhibitors suppressed the differentiation of Th0 cells into Th1 and Th2 cells (Figure 2K,L). Bone marrow‐derived mesenchymal stem cell‐induced and activated dendritic cells were used to activate CD4^+^ T cells for mixed lymphocyte culture assays. Consistent with the results from anti‐CD3/CD28 antibodies‐stimulated CD4^+^ T cells, proliferation and Tcf1 expression in CD4^+^ T cells treated with MAPK13‐inhibitors were suppressed (Figure S3A,B).

### 2.3 Deletion of Mapk13 in T Cells Reduces CAV

**FIGURE 3:**
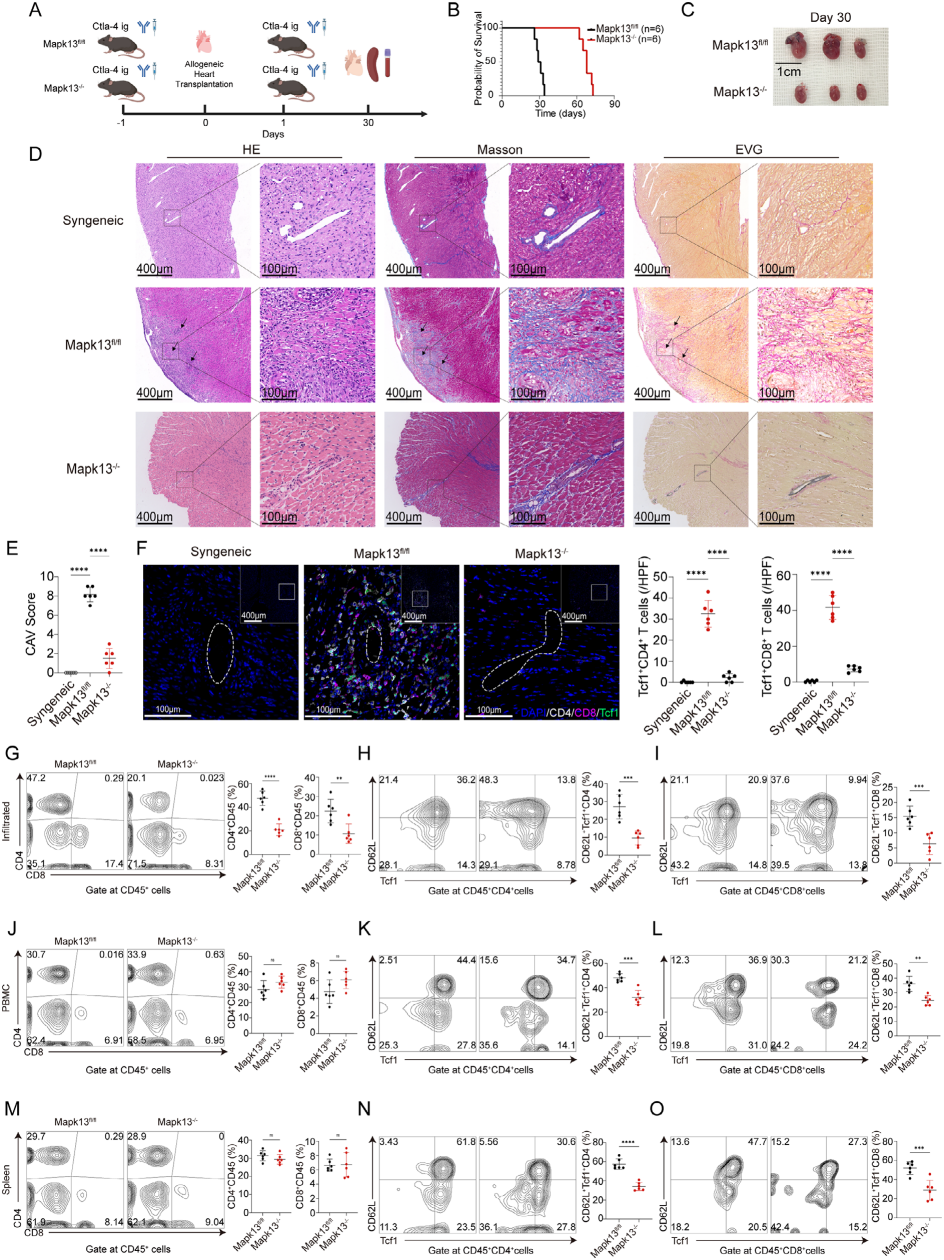
Mapk13 deletion reduces CAV. (A) Construction of the CAV model in Mapk13^fl/fl^ and Mapk13^−/−^ recipient mice. (B) Survival curves of transplanted hearts in syngeneic, Mapk13^fl/fl^ and Mapk13^−/−^ recipient mice (*n* = 6). (C–F) Transplanted hearts from syngeneic, Mapk13^fl/fl^ and Mapk13^−/−^ recipient mice were harvested on day 30 post‐heart transplantation. Morphological comparisons (C), representative histological staining (D), chronic allograft vasculopathy (CAV) scores (E), and representative mIHC staining (F) of transplanted hearts (*n* = 6). (G–O) Representative flow cytometric plots of CD4^+^ T cells and CD8^+^ T cells, as well as overlay histograms of CD62l^+^Tcf1^+^CD4^+^T cells and CD62l^+^Tcf1^+^CD8^+^T cells, along with their proportions, from transplanted hearts, peripheral blood, and spleens of Mapk13^fl/fl^ and Mapk13^−/−^ recipient mice (*n* = 6). ns, not significant, ^*^ *p* < 0.01, ^**^ *p* < 0.001, ^****^ *p* < 0.00001.

Consistent with previous studies, depletion of Cd4^+^ or Cd8^+^ T cells prior to CAV induction revealed that Cd4^+^ T cells, rather than Cd8^+^ T cells, play a dominant role in the development of CAV (Figure S4). Since Mapk13 inhibition reduces Tcf1 phosphorylation in CD4^+^ T cells, we generated Mapk13^−/−^ mice to investigate the impact of Mapk13 deletion on CAV. Hearts from BALB/c mice were heterotopically transplanted into the abdomens of Mapk13^fl/fl^ and Mapk13^−/−^ recipient mice. To establish the CAV model, recipient mice received 10 mg/kg Ctla4 Ig intraperitoneally 1 day before and after transplantation (Figure 3A). Transplanted hearts in Mapk13^fl/fl^ recipient mice ceased beating between days 26 and 34, whereas those in Mapk13^−/−^ mice remained functional until days 62–73 (Figure 3B). Transplanted hearts harvested on day 30 after transplantation of Mapk13^−/−^ recipient mice were notably smaller compared with those of Mapk13^fl/fl^ recipient mice (Figure 3C). In Mapk13^fl/fl^ recipient mice, transplanted hearts exhibited diffuse mononuclear cell infiltration, with focal accumulation around coronary arteries. In contrast, Mapk13^−/−^ recipient mice displayed significantly reduced mononuclear cell infiltration (Figure 3D). Transplanted hearts in Mapk13^fl/fl^ recipient mice exhibited extensive fibrous tissue hyperplasia and centripetal coronary artery narrowing, accompanied by yellow lipid plaque deposition in the intima (Figure 3D). In contrast, transplanted hearts from Mapk13^−/−^ recipient mice exhibited significantly reduced fibrous tissue hyperplasia, with minimal thickening and narrowing of the coronary lumen (Figure 3D). Although some arteries exhibited intimal hyperplasia and elastic fiber damage, the majority of coronary lumens and fibers maintained normal structural integrity (Figure 3D). CAV severity in transplanted hearts was assessed based on interstitial inflammation (i), vascular fibrous intimal thickening (v), and the percentage of vascular fibrous intimal thickening (pv) (Table 1) [1]. Transplanted hearts of Mapk13^−/−^ recipient mice exhibited significantly lower CAV scores compared to those of Mapk13^fl/fl^ recipient mice (Figure 3E). CD4^+^ T cells and CD8^+^ T cells prominently aggregated around coronary arteries, contributing to CAV in transplanted hearts of Mapk13^fl/fl^ recipient mice. The infiltrating T cells in the transplanted hearts of Mapk13^−/−^ recipient mice were significantly reduced compared with those of Mapk13^fl/fl^ recipient mice. Furthermore, Tcf1^+^ T cells were also significantly reduced in the transplanted hearts of Mapk13^−/−^ recipient mice (Figure 3F). The proportions of CD4^+^, CD8^+^, CD62l^+^Tcf1^+^CD4^+^, and CD62l^+^Tcf1^+^CD8^+^ T cells infiltrating the transplanted hearts were significantly lower in Mapk13^−/−^ recipient mice than in Mapk13^fl/fl^ mice (Figure 3G–I; Figure S5A). The absolute numbers of infiltrating CD4^+^ T cells and Tcf1^+^CD4^+^ T cells in transplanted hearts were significantly reduced in Mapk13^−/−^ recipient mice compared with Mapk13^fl/fl^ mice (Figure S5B). No significant differences were found in the overall proportions of CD4^+^ and CD8^+^ T cells in the peripheral blood or spleen, whereas CD62l^+^Tcf1^+^CD4^+^ and CD62l^+^Tcf1^+^CD8^+^ T cells were reduced in Mapk13^−/−^ recipients (Figure 3J–O).

**TABLE 1:** Chronic allograft vasculopathy scores.

CAV score	Abbreviation	0	1	2	3
interstitial inflammation	I	<10%	10%–25%	26%–50%	>50%
vascular fibrous intimal thickening	V	None	≤25%	26%–50%	>50% reduction in luminal area
percentage of vascular fibrous intimal thickening	Pv	<10%	10%–25%	26%–50%	>50%

### 2.4 Tcf1 Positively Regulates the Transcription of *Slc7a5*

**FIGURE 4:**
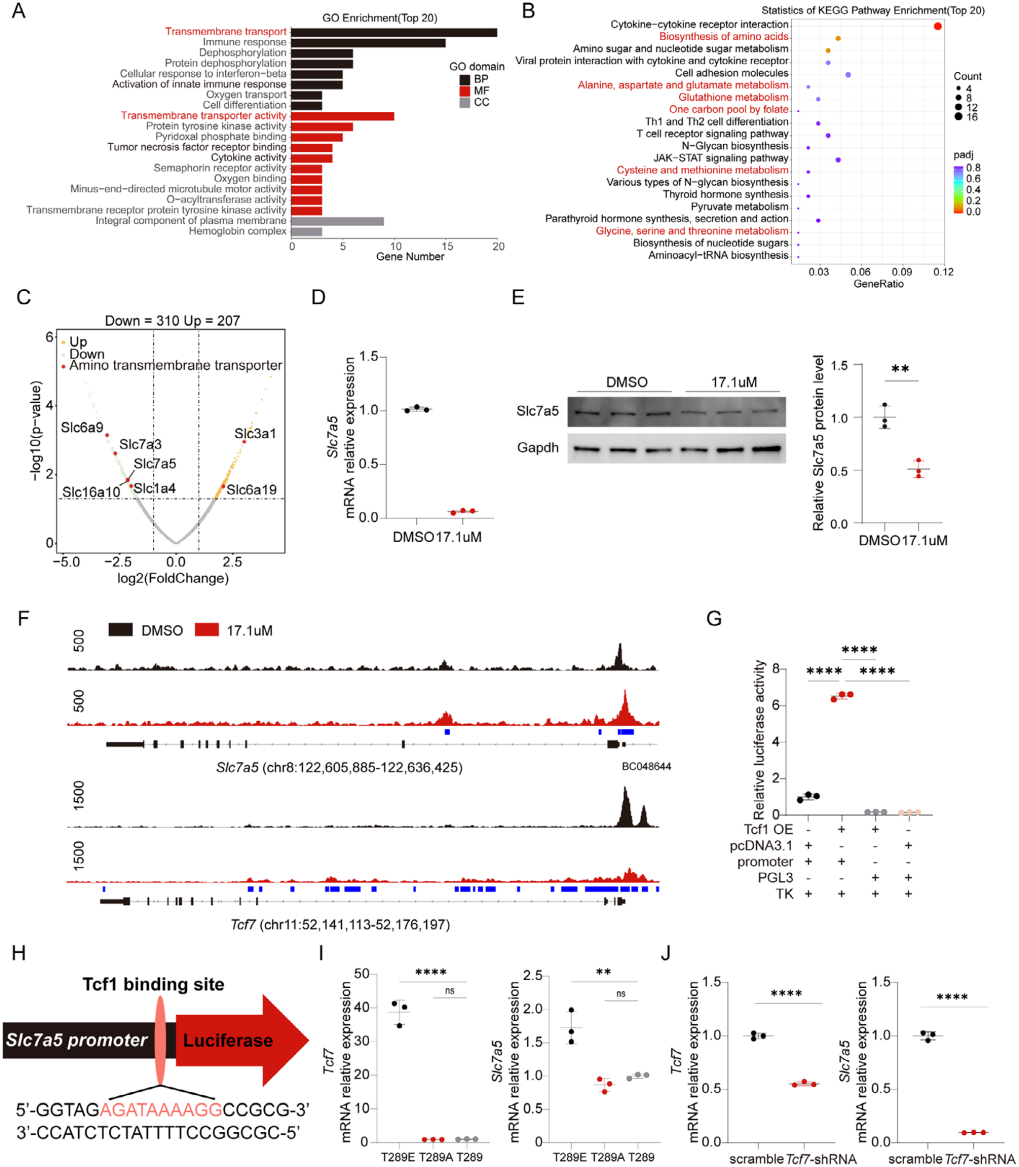
Tcf1 positively regulates *Slc7a5* transcription. (A–G) CD4^+^ T cells were isolated from the spleen and stimulated with anti‐CD3/CD28 antibodies in the presence of MAPK13‐inhibitors (17.1 µm) or DMSO for 72 h. The cells were then collected for RNA‐seq, RT‐qPCR, western blotting, and ChIP‐seq. (A) Gene Ontology enrichment analysis showing the top 20 downregulated terms ranked by gene ratio. (B) KEGG pathway enrichment analysis. (C) Volcano plot of RNA‐seq results displaying upregulated and downregulated genes. (D) Relative mRNA expression levels of *Tcf7* and *Slc7a5* (*n* = 3). (E) Protein levels of Slc7a5 (*n* = 3). (F) ChIP‐seq tracks of Tcf1 binding at *Slc7a5 and Tcf7* loci. Differential binding analysis was performed using MACS2. (G) Dual‐luciferase assay detecting Tcf1 binding to a 100‐bp promoter fragment of *Slc7a5* (*n* = 3). (H) Predicted Tcf1 binding site in the *Slc7a5* promoter fragment identified using JASPAR. (I). Splenic CD4^+^ T cells stimulated with anti‐CD3/CD28 antibodies for 24 h were infected with lentivirus carrying murine pCDH‐Tcf1^T289E^, pCDH‐Tcf1^T289A^ or pCDH‐Tcf1. CD4^+^ T cells were collected 48 h post‐infection, and RT‐qPCR was performed to detect the mRNA expression levels of *Tcf7* and *Slc7a5* (*n* = 3). (J) Splenic CD4^+^ T cells stimulated with anti‐CD3/CD28 antibodies for 24 h were infected with pCDH‐H1‐EF1‐Puro scramble control or murine Tcf1 shRNA retroviruses. RT‐qPCR was performed to detect the mRNA expression levels of *Tcf7* and *Slc7a5* (*n* = 3). ^**^ *p* < 0.001, ^***^ *p* < 0.0001, ^****^ *p* < 0.00001.

Tcf1 exhibits transcription factor activity, modulating gene expression by binding to the promoter region. To investigate how CD4^+^ T cells maintain their stem cell‐like properties, we conducted RNA‐seq to analyze gene expression differences between MAPK13‐inhibitors‐treated and DMSO‐treated cells. Among 517 differentially expressed genes, 310 were downregulated in MAPK13‐inhibitors‐treated CD4^+^ T cells. Enrichment analysis of biological processes and molecular functions revealed a downregulation in transmembrane transport (Figure 4A). Pathway enrichment analysis identified a downregulation in the metabolism of various amino acids (Figure 4B). The volcano plot displayed downregulation of amino acid transporter genes *Slc6a9*, *Slc7a3*, *Slc7a5*, *Slc6a10*, and *Slc1a4*, along with an upregulation of *Slc3a1* and *Slc6a19* (Figure 4C). Previous studies suggest that the amino acid transporters Slc7a5, Slc38a1, Slc38a2, and Slc43a2 are involved in methionine transport, with Slc7a5 being the most abundant in activated CD4^+^ T cells [12, 13]. Additionally, RT‐qPCR analysis confirmed a significant downregulation of *Tcf7* and *Slc7a5* mRNA expression in CD4^+^ T cells treated with the MAPK13‐inhibitors (Figure 4D). Consistent with mRNA expression levels, a significant reduction in Slc7a5 protein levels was observed in CD4^+^ T cells treated with MAPK13‐inhibitors (Figure 4E). To investigate the regulation of *Slc7a5* transcription by Tcf1, we performed ChIP‐seq using anti‐Tcf1 antibodies. In cells treated with MAPK13‐inhibitors, Tcf1 binding to the promoter regions of *Slc7a5*, *Tcf7*, and *Lef1* was markedly reduced, suggesting that Tcf1 promotes *Slc7a5* transcription, consistent with the decreased *Slc7a5* mRNA expression (Figure 4F). Moreover, Tcf1 does not bind to the promoter regions of *Slc1a4*, *Slc16a10, Slc6a9*, and *Slc7a3* (Figure S6). Based on ChIP‐seq results indicating potential Tcf1 binding sites in the *Slc7a5* promoter, a 100‐bp fragment was designed and cloned into a PGL3 plasmid containing the firefly luciferase gene. Dual‐luciferase assays confirmed Tcf1 binding to the 100‐bp fragment of the *Slc7a5* promoter (Figure 4G). Additionally, motif prediction using the JASPAR database identified the most probable Tcf1 binding motif in the *Slc7a5* promoter region, matching the sequence “AGATAAAAGG” with a relative score of 0.888 (Figure 4H). To verify the binding of p‐Tcf1 to the *Slc7a5* promoter, we constructed pCDH‐Tcf1 plasmids. The pCDH‐Tcf1 plasmid was constructed for Tcf1 at T289. The threonine codon “ACA” was mutated to glutamic acid (E) “GAA” to mimic phosphorylation (pCDH‐Tcf1^T289E^), and to alanine (A) “GCA” to mimic dephosphorylation (pCDH‐Tcf1^T289A^). CD4^+^ T cells were infected with pCDH‐Tcf1^T289E^, pCDH‐Tcf1^T289A^, or pCDH‐Tcf1 lentiviruses. RT‐qPCR analysis revealed that compared to CD4^+^ T cells infected with lentivirus carrying pCDH‐Tcf1^T289A^ or pCDH‐Tcf1, CD4^+^ T cells infected with lentivirus carrying pCDH‐Tcf1^T289E^ exhibited significantly higher expression levels of *Tcf7* and *Slc7a5* (Figure 4I). In addition, CD4^+^ T cells were infected with pCDH‐H1‐EF1‐Puro (scramble) or murine Tcf1 shRNA retroviruses. RT‐qPCR analysis revealed that knockdown of *Tcf7* mRNA led to a significant downregulation of both *Tcf7* and *Slc7a5* mRNA (Figure 4J). These findings demonstrated that in Tcf1^+^CD4^+^ T cells, p‐Tcf1 promotes *Slc7a5* transcription by binding directly to the *Slc7a5* promoter region.

### 2.5 Deletion of Slc7a5 in T Cells Induces Chronic Graft Tolerance

**FIGURE 5:**
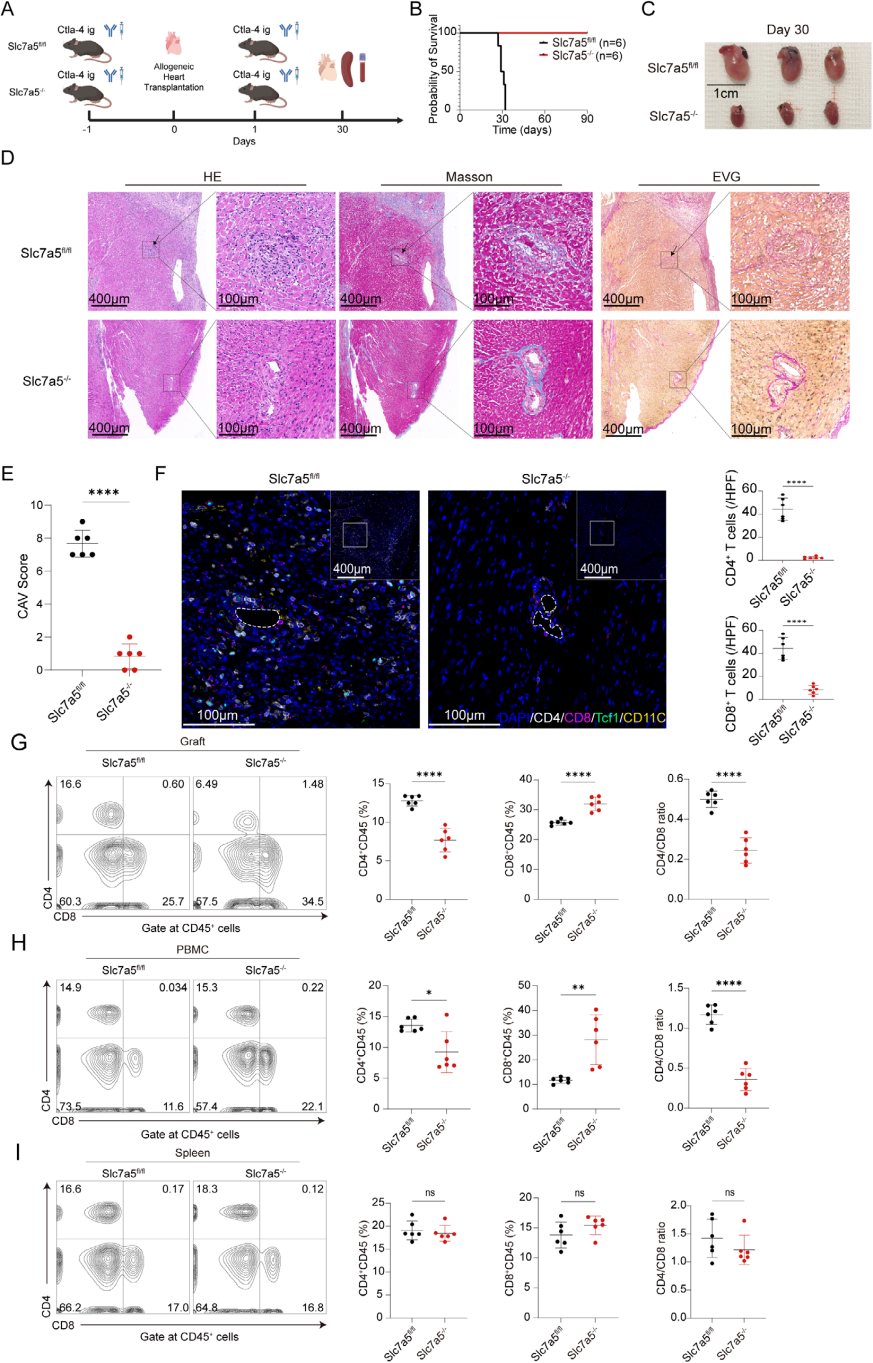
Slc7a5 deletion induces chronic graft tolerance. (A) Construction of the CAV model in Slc7a5^fl/fl^ and Slc7a5^−/−^ recipient mice. (B) Survival curves of transplanted hearts in Slc7a5^fl/fl^ and Slc7a5^−/−^ recipient mice (*n* = 6). (C–F) Transplanted hearts from Slc7a5^fl/fl^ and Slc7a5^−/−^ recipient mice were harvested on day 30 post‐heart transplantation. Morphological comparisons (C), representative histological staining (D), CAV scores (E), and representative mIHC staining (F) of transplanted hearts (*n* = 6). (G–I) Representative flow cytometric plots and proportions of CD4^+^ T cells and CD8^+^ T cells in transplanted hearts, peripheral blood, and spleens of Slc7a5^fl/fl^ and Slc7a5^−/−^ recipient mice (*n* = 6). ns, not significant, ^**^ *p* < 0.001, ^****^ *p* < 0.00001.

To evaluate the role of Slc7a5 in CD4^+^ T cells in the context of CAV, we generated Slc7a5^−/−^ mice to establish the CAV model (Figure 5A). In Slc7a5^fl/fl^ recipient mice, transplanted hearts ceased beating between days 27 and 32. In contrast, transplanted hearts in Slc7a5^−/−^ mice remained functional with continuous beating (Figure 5B). On day 30 post‐transplantation, the transplanted hearts in Slc7a5^−/−^ mice were significantly smaller compared with those in Slc7a5^fl/fl^ recipient mice (Figure 5C). Rare mononuclear cell infiltration and intact elastic fibers in the arterial walls, with no evidence of fibrous tissue hyperplasia and narrowing of the coronary artery lumen were observed in transplanted hearts of Slc7a5^−/−^ recipient mice (Figure 5D). Correspondingly, CAV scores of transplanted hearts of Slc7a5^−/−^ recipient mice were significantly lower than those in Slc7a5^fl/fl^ mice (Figure 5E). The infiltrating T cells and dendritic cells in the transplanted hearts of Slc7a5^−/−^ recipient mice were significantly reduced compared with those of Slc7a5^fl/fl^ recipient mice. Furthermore, Tcf1^+^ T cells were also significantly reduced in the transplanted hearts of Slc7a5^−/−^ recipient mice (Figure 5F). In the transplanted hearts and in peripheral blood, the proportion of CD4^+^ T cells was significantly reduced, while the proportion of CD8^+^ T cells increased, resulting in a significant decrease in the CD4/CD8 ratio in Slc7a5^−/−^ recipient mice compared with those in Slc7a5^fl/fl^ recipient mice (Figure 5G,H). However, no significant difference in the proportion of CD4^+^ and CD8^+^ T cells were detected in the spleen (Figure 5I).

To determine whether the Mapk13‐Tcf1‐Slc7a5 axis regulates chronic allograft rejection in a CD4^+^ T cell‐intrinsic manner, we performed adoptive transfer experiments using Rag1 knockout recipient mice. CD4^+^ T cells were isolated from the spleens of wild‐type, Mapk13^−/−^, or Slc7a5^−/−^ mice following establishment of a chronic cardiac allograft rejection model and intravenously transferred into Rag1 knockout mice. Recipient mice subsequently underwent heterotopic abdominal heart transplantation using BALB/c donor hearts (Figure S7A). Rag1 knockout mice reconstituted with wild‐type CD4^+^ T cells exhibited rapid graft deterioration, with complete cessation of cardiac beating occurring between days 12 and 14 post‐transplantation. In contrast, transplanted hearts in Rag1 knockout mice reconstituted with Mapk13^−/−^ or Slc7a5^−/−^ CD4^+^ T cells maintained sustained beating over the same observation period. Prolonged allograft survival accompanied by reduced CAV severity in Rag1 knockout mice reconstituted with Mapk13^−/−^ or Slc7a5^−/−^ CD4^+^ T cells is consistent with a role for the Mapk13‐Tcf1‐Slc7a5 axis within CD4^+^ T cells in modulating pathogenic alloimmune responses during chronic cardiac allograft rejection (Figure S7B,C).

### 2.6 Methionine Restriction Impairs the Stemness of CD4^+^ T Cells

**FIGURE 6:**
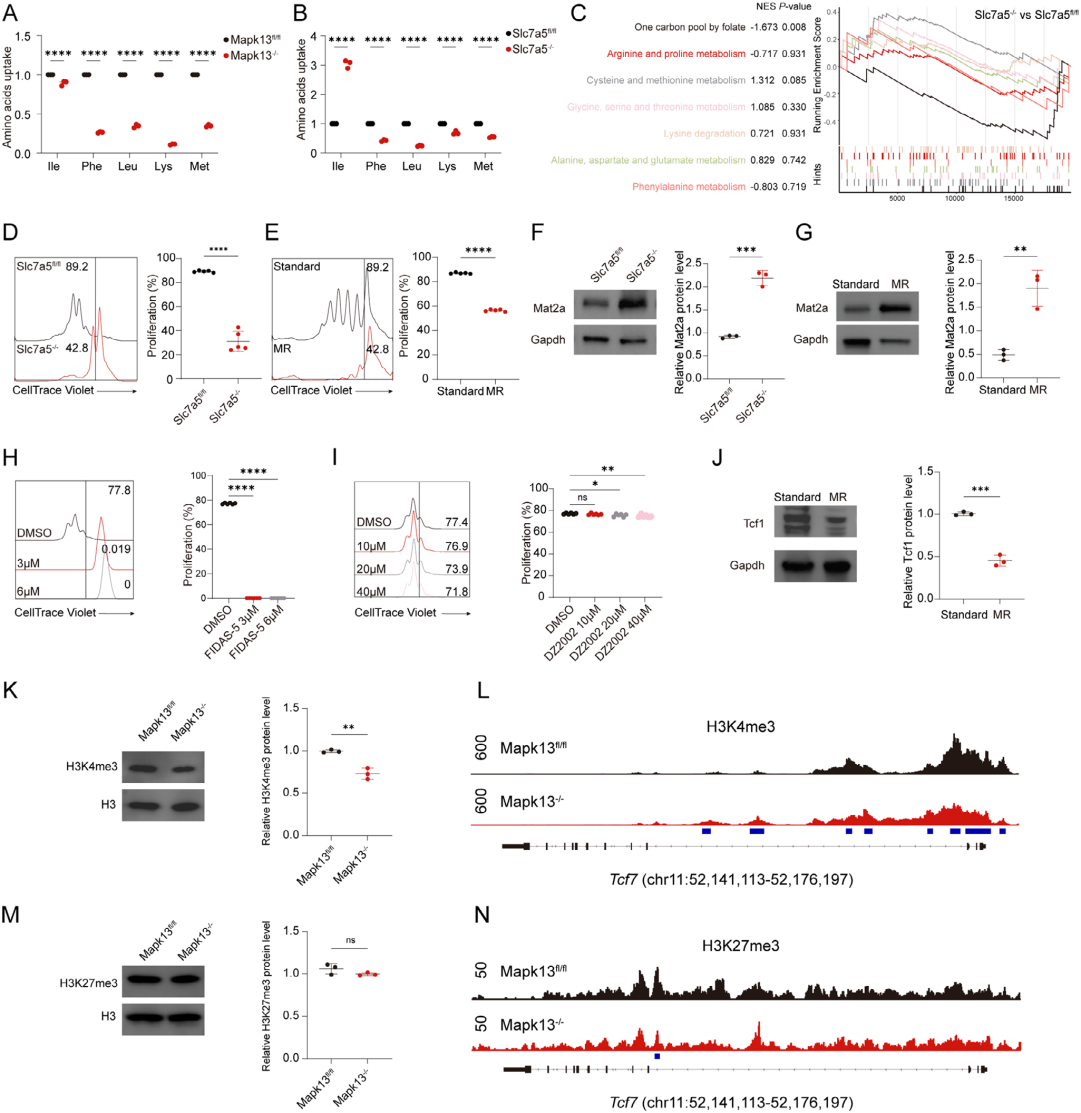
Methionine restriction impairs the stem‐like properties of CD4^+^ T cells. (A,B) Effects of Mapk13 deletion and Slc7a5 deletion on amino acid consumption from the culture media in CD4^+^ T cells (*n* = 3). (C) CD4^+^ T cells were isolated from the spleen of Slc7a5^fl/fl^ and Slc7a5^−/−^ mice and stimulated with anti‐CD3/CD28 antibodies for 72 h. Cells were subsequently collected for RNA‐seq. Gene Set Enrichment Analysis (GSEA) of amino acid metabolism‐related KEGG signaling pathways (*n* = 3). (D‐K) CD4^+^ T cells were isolated from the spleen and stimulated with anti‐CD3/CD28 antibodies for 72 h. Then the cells were collected for Western blotting or flow cytometry. (D) Cell proliferation of CD4^+^ T cells from Slc7a5^fl/fl^ and Slc7a5^−/−^ mice (*n* = 6). (E) Cell proliferation of CD4^+^ T cells cultured in standard medium or MR medium (*n* = 5). (F) Mat2a protein levels of CD4^+^ T cells from Slc7a5^fl/fl^ and Slc7a5^−/−^ mice (*n* = 3). (G) Mat2a protein levels of CD4^+^ T cells cultured in standard medium or MR medium (*n* = 3). (H) Cell proliferation of CD4^+^ T cells cultured in the presence of FIDAS‐5 or DMSO (*n* = 5). (I) Cell proliferation of CD4^+^ T cells cultured in the presence of DZ2002 or DMSO (*n* = 5). (J) Tcf1 protein levels of CD4^+^ T cells cultured in standard medium or MR medium (*n* = 3). (K) Protein levels of H3K4me3 in CD4^+^ T cells from Mapk13^fl/fl^ and Mapk13^−/−^ mice (*n* = 3). (L) CUT&Tag‐seq tracks of H3K4me3 binding at *Tcf7* loci. (M) Protein levels of H3K27me3 in CD4^+^ T cells from Mapk13^fl/fl^ and Mapk13^−/−^ mice (*n* = 3). (N) CUT&Tag‐seq tracks of H3K27me3 binding at *Tcf7* loci. ns, not significant, ^*^ *p* < 0.01, ^**^ *p* < 0.001, ^***^ *p* < 0.0001, ^****^ *p* < 0.00001.

To assess whether deletion of Mapk13 or Slc7a5 affected stem‐like properties of CD4^+^ T cells, splenic CD4^+^ T cells isolated from WT, Mapk13^−/−^, and Slc7a5^−/−^ mice were stimulated in vitro. Ly108^+^ cells largely overlapped with the Tcf1^+^ population and exhibited superior proliferative and differentiation capacities compared with Ly108^−^ cells (Figure S8A,B). Notably, the functional potency of stem‐like CD4^+^ T cells was progressively diminished in Mapk13^−/−^ and Slc7a5^−/−^ mice, consistent with impaired T cell stemness upon disruption of the Mapk13‐Tcf1‐Slc7a5 axis. To evaluate the ability of CD4^+^ T cells to uptake amino acids, mass spectrometry was employed to measure amino acid concentrations in the culture medium. CD4^+^ T cells from Mapk13^−/−^ mice exhibited significantly reduced amino acid consumption from the culture media of phenylalanine, leucine, lysine, and methionine compared to those from Mapk13^fl/fl^ mice (Figure 6A). Similarly, CD4^+^ T cells from Slc7a5^−/−^ mice demonstrated a significant reduction in the amino acid consumption from the culture media of these amino acids compared to those from Slc7a5^fl/fl^ mice (Figure 6B). Gene set enrichment analysis of amino acid metabolism‐related KEGG signaling pathways revealed significant downregulation of the “One‐carbon pool by folate” pathway in CD4^+^ T cells from Slc7a5^−/−^ mice compared to those from Slc7a5^fl/fl^ mice, suggesting inhibition of the methionine cycle pathway. (Figure 6C). Proliferation of CD4^+^ T cells from Slc7a5^−/−^ mice was significantly reduced compared to that in cells from Slc7a5^fl/fl^ mice (Figure 6D). A similar reduction in cell proliferation was observed in CD4^+^ T cells cultured in methionine‐restricted (MR) medium, as compared to cells grown in standard medium (Figure 6E). Consistent with the findings from anti‐CD3/CD28 antibody‐stimulated CD4^+^ T cells, proliferation was suppressed in CD4^+^ T cells from Slc7a5^−/−^ mice and those cultured in MR medium in the mixed lymphocyte culture assay (Figure S9A,B). Mat2a, a key enzyme in the intracellular methionine cycle, accumulates in methionine‐deficient CD4^+^ T cells, which enhances sensitivity to methionine and promotes its uptake [14]. Protein levels of Mat2a were significantly higher in CD4^+^ T cells from Slc7a5^−/−^ mice compared to those from Slc7a5^fl/fl^ mice (Figure 6F). Likewise, CD4^+^ T cells cultured in MR medium exhibited increased Mat2a accumulation (Figure 6G). Inhibition of Mat2a using FIDAS‐5 in standard medium resulted in a significant reduction in CD4^+^ T cell proliferation (Figure 6H). However, inhibiting S‐adenosylhomocysteine hydrolase with DZ2002, which prevents the demethylation of adenosylmethionine (SAM) back to methionine, did not impact CD4^+^ T cell proliferation (Figure 6I). Consistent with the results from anti‐CD3/CD28 antibody‐stimulated CD4^+^ T cells, proliferation was suppressed in CD4^+^ T cells from Slc7a5^−/−^ mice and those cultured in MR medium in the mixed lymphocyte culture assay (Figure S9C,D). As SAM serves as a critical methyl donor, inhibiting its production affects histone modifications [15, 16]. Interestingly, Tcf1 protein levels were reduced in CD4^+^ T cells cultured in MR medium compared to those in standard medium (Figure 6J). H3K4me3 and H3K27me3 are selectively modified in ways that are associated with the expression of genes critical for the effector or stem cell programs of T cells [17]. H3K4me3 and H3K27me3 protein levels were examined in CD4^+^ T cells isolated from Mapk13^−/−^ and Mapk13^fl/fl^ mice. CUT&Tag‐seq assays specific for these histone marks were further performed on the same cells. The H3K4me3 protein level was significantly reduced in Mapk13^−/−^ CD4^+^ T cells, while H3K27me3 remained unchanged (Figure 6K,M). Consistently, CUT&Tag‐seq analysis showed reduced H3K4me3 enrichment at the *Tcf7* promoter in Mapk13^−/−^ CD4^+^ T cells, whereas H3K27me3 enrichment at this locus was unaffected (Figure 6L,N).

### 2.7 MR Diet Induces Chronic Graft Tolerance

**FIGURE 7:**
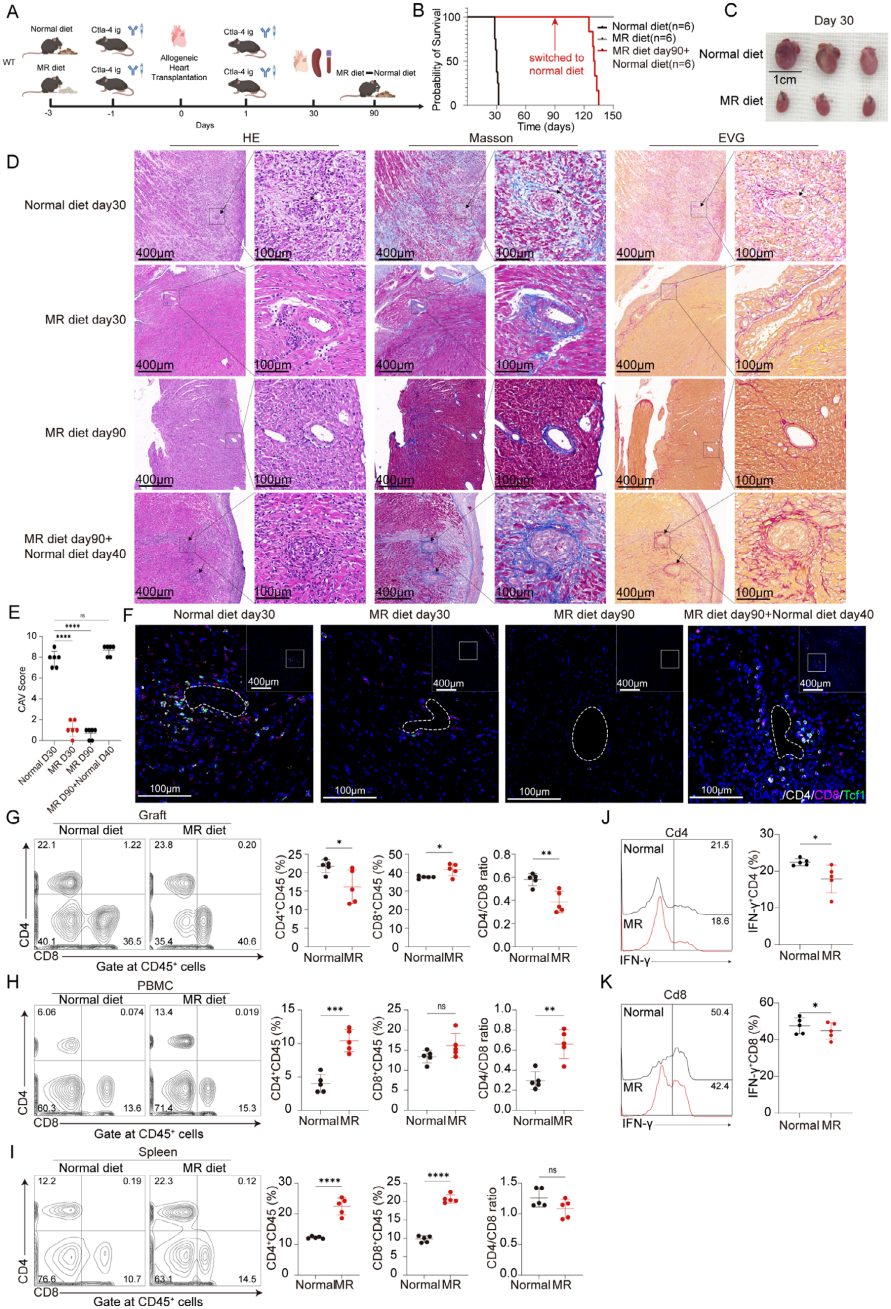
MR diet induces graft tolerance. (A) Construction of the CAV model in normal diet and MR diet recipient mice. (B) Survival curves of transplanted hearts in recipient mice on a normal diet, an MR diet, or an MR diet switched to a normal diet on day 90 (*n* = 6). (C–F) Transplanted hearts from normal diet and MR diet recipient mice were harvested on day 30 post‐heart transplantation. Morphological comparisons (C), histological stain (D), CAV scores (E), and mIHC stain (F) (*n* = 6). (G–I) Representative flow plots and proportions of CD4^+^ T cells and CD8^+^ T cells in transplanted hearts, peripheral blood, and spleens of recipient mice on a normal diet or a MR diet (*n* = 5). (J,K). Lymphocytes infiltrating the transplanted hearts were isolated and then stimulated with phorbol 12‐myristate 13‐acetate containing brefeldin A for 4.5 h. Representative flow cytometric overlay histograms and proportions of IFN‐γ^+^CD4 cells and IFN‐γ^+^CD8 cells in recipient mice on a normal diet or a MR diet (*n* = 5). ns, not significant, ^*^ *p* < 0.01, ^**^ *p* < 0.001, ^***^ *p* < 0.0001, ^****^ *p* < 0.00001.

We hypothesized that methionine restriction would impair CD4^+^ T cell stemness, thereby inducing immune tolerance in transplanted hearts. Therefore, the CAV model was constructed using mice fed either a normal diet or an MR diet (Figure 7A). Notably, recipient mice on the MR diet exhibited significantly lower body weight compared to those on the normal diet (Figure S10), while their transplanted hearts maintained stable and continuous beating (Figure 7B). Interestingly, when the MR diet was replaced with a normal diet on day 90 post‐transplantation, heart grafts ceased beating between days 35 and 45 after the dietary switch (Figure 7B). On day 30 post‐transplantation, transplanted hearts from mice on the MR diet were significantly smaller than those from mice on the normal diet (Figure 7C). Transplanted hearts of recipient mice on the MR diet exhibited rare mononuclear cell infiltration on day 30 post‐transplantation. Mononuclear cell infiltration was markedly reduced in transplanted hearts of recipient mice on the MR diet on day 90 post‐transplantation. In contrast, on day 40 post‐switch to the normal diet, significant mononuclear cell infiltration was observed in the transplanted hearts (Figure 7D). Masson and EVG staining revealed intact elastic fibers in arterial walls with no evidence of fibrous tissue hyperplasia or coronary artery narrowing in transplanted hearts of recipient mice on the MR diet, either on day 30 or 90 post‐transplantation (Figure 7D). However, on day 40 post‐switch to the normal diet, the transplanted hearts exhibited severe fibrous tissue hyperplasia, concentric narrowing, and occlusion of the coronary arteries (Figure 7D). The CAV scores of transplanted hearts from recipient mice on the MR diet were significantly lower compared to those from mice switched to the normal diet (Figure 7E). mIHC staining showed reduced infiltration of T cells, Tcf1^+^ T cells, and dendritic cells in transplanted hearts of recipient mice on the MR diet compared to those switched to the normal diet (Figure 7F; Figure S11). Flow cytometry analysis of infiltrating mononuclear cells in the transplanted hearts, peripheral blood, and spleens of recipient mice on either diet at day 30 post‐heart transplantation revealed that in the transplanted hearts of mice on the MR diet, the proportion of infiltrating CD4^+^ T cells was significantly reduced, while the proportion of CD8^+^ T cells relatively increased, leading to a significant decrease in the CD4/CD8 ratio (Figure 7G). In the peripheral blood of recipient mice on the MR diet, the proportion of CD4^+^ T cells increased, while the proportion of CD8^+^ T cells remained unchanged, resulting in a significant increase in the CD4/CD8 ratio (Figure 7H). In the peripheral blood, both CD4^+^ and CD8^+^ T cells increased without a significant difference in the CD4/CD8 ratio (Figure 7I). Additionally, CD4^+^ and CD8^+^ T cells infiltrating the transplanted hearts of recipient mice on the MR diet exhibited significantly reduced IFN‐γ production (Figure 7J,K).

### 2.8 MAPK13‐Inhibitors Alleviate Chronic Graft Arteriosclerosis

**FIGURE 8:**
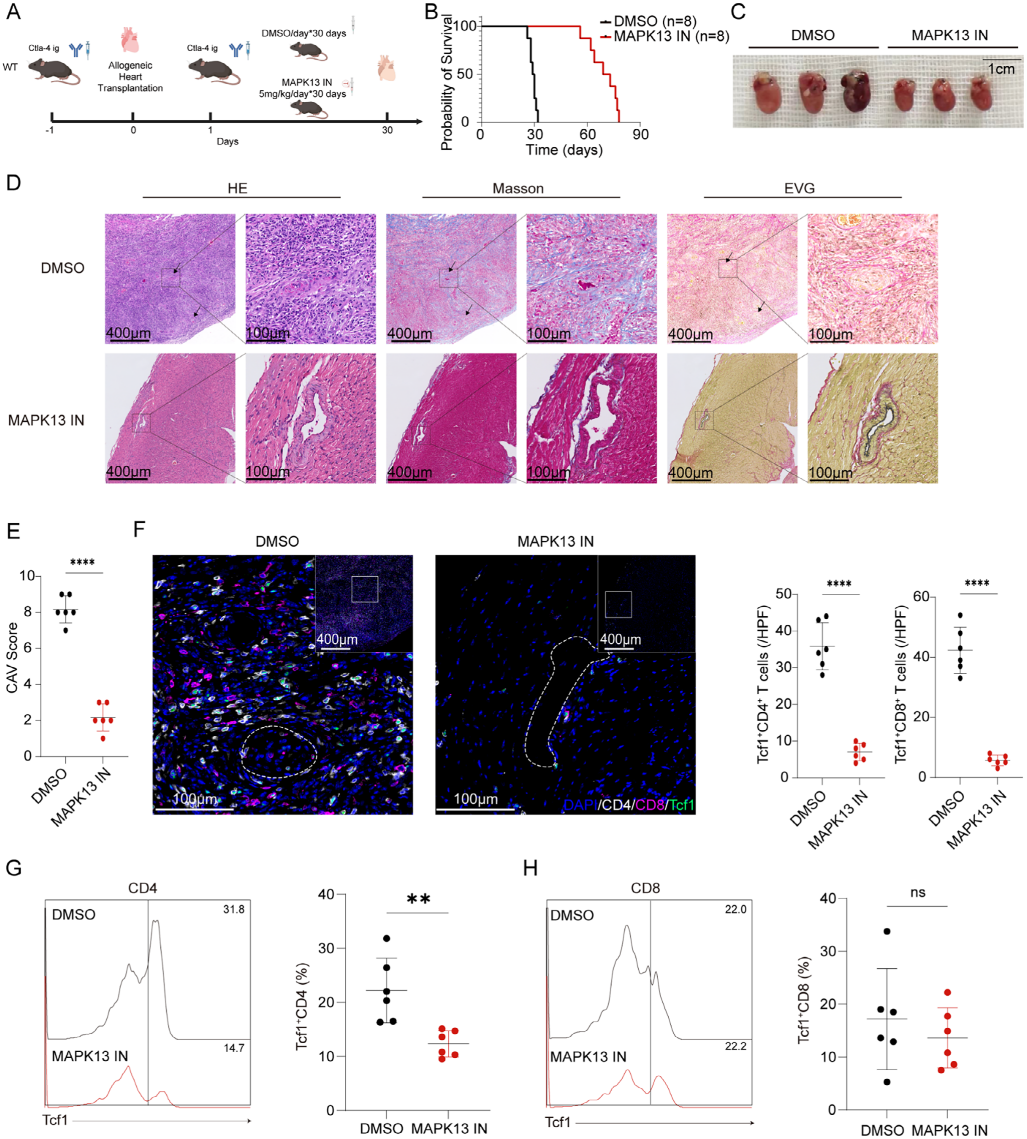
Mapk13 inhibition alleviates CAV. (A) Construction of the CAV model in MAPK13‐inhibitors and DMSO treated recipient mice. (B) Survival curves of transplanted hearts in MAPK13‐inhibitors‐treated and DMSO‐treated recipient mice (*n* = 8). (C–F) Transplanted hearts from MAPK13‐inhibitors‐treated and DMSO‐treated recipient mice were harvested on day 30 post‐heart transplantation. Morphological comparisons (C), histological stain (D), CAV scores (E), and mIHC staining (F) (*n* = 6). (G,H) Representative flow cytometric overlay histograms and proportions of Tcf1^+^CD4 T cells and Tcf1^+^CD8 T cells in transplanted hearts of recipient mice treated with MAPK13‐inhibitors or DMSO (*n* = 6). ns, not significant, ^**^ *p* < 0.001, ^****^ *p* < 0.00001.

To evaluate the potential of MAPK13 inhibition in prolonging graft survival, we established the CAV model through intraperitoneal injection of MAPK13‐inhibitors. After heart transplantation, recipient mice in the MAPK13‐inhibitors group received 5 mg/kg/day of MAPK13‐inhibitors for 30 days, while the control group received an equivalent dose of DMSO for 30 days (Figure 8A). Transplanted hearts in the control group ceased beating between days 26 and 32, while those in the MAPK13‐inhibitors group continued to beat until days 56 to 78 (Figure 8B). Transplanted hearts were collected for analysis on day 30 post‐transplantation. Hearts in the MAPK13‐inhibitors group were significantly smaller than those in the control group (Figure 8C). Mononuclear cell infiltration and fibrous tissue hyperplasia in the transplanted hearts of the MAPK13‐inhibitors group were significantly reduced compared to the control group (Figure 8D). No significant coronary artery narrowing was observed in the transplanted hearts of the MAPK13‐inhibitors group compared to those in the control group (Figure 8D). The CAV scores of transplanted hearts were significantly lower in the MAPK13‐inhibitors group (Figure 8E). Infiltration of CD4^+^ and CD8^+^ T cells, as well as Tcf1^+^CD4^+^ T cells, was significantly reduced in the MAPK13‐inhibitors group compared to the control group (Figure 8F). Compared to the control group, the proportion of Tcf1^+^CD4^+^ T cells in the transplanted hearts decreased in the MAPK13‐inhibitors group, while the proportion of Tcf1^+^CD8^+^ T cells remained unchanged (Figure 8G,H). Similarly, in an allogeneic kidney transplantation model, recipient mice maintained on an MR diet exhibited significantly attenuated CAV severity compared with recipients fed a normal chow diet (Figure S12).

## 3 Conclusion

Recent advances underscore the pivotal role of stem‐like T cells in chronic allograft vasculopathy. In contrast to the goal of enhancing tumor‐infiltrating stem‐like T cells, reducing graft arterial damage necessitates the depletion of stem‐like T cells. Stem‐like Tcf1^+^CD4^+^ T cells in grafts are associated with CAV and long‐term graft survival [18]. Tcf1 is a critical regulator of T cell fate and development, enabling T cells to self‐renew and differentiate within resident tissues. Current research on graft rejection predominantly focuses on strategies to inhibit effector cells. However, the approach for targeting Tcf1^+^CD4^+^ T cells to mitigate CAV remains unclear. In this study, we discovered that Mapk13 phosphorylates Tcf1 at T289. Additionally, we found that Tcf1 binds to its own promoter, contributing to the persistence of chronic graft damage.

Stem cell‐like T cells require substantial protein consumption for self‐renewal and proliferative differentiation, with increased expression of amino acid transporters to facilitate amino acid uptake from the environment [13, 19]. Previous studies suggest that amino acid uptake during CD4^+^ T cell activation is primarily dependent on Slc7a5 [20, 21]. While Slc7a5 mediates the uptake of multiple essential amino acids, our findings suggest that methionine represents a key downstream metabolic node linking Slc7a5 activity to epigenetic control of CD4^+^ T cell stemness. Rather than acting as a unique substrate of Slc7a5, methionine appears to function as a critical metabolic effector whose availability integrates amino acid transport with one‐carbon metabolism and histone methylation.

Limiting methionine metabolism in naive T cells preserves their stemness; however, its impact on the stemness of T cells following TCR activation remains unclear [16]. The intracellular methionine pool in activated T cells is almost entirely derived from extracellular methionine and is highly sensitive to fluctuations in extracellular methionine concentration. Our research shows that upon TCR activation, CD4^+^ T cells enhance methionine uptake and reprogram methionine metabolism to facilitate T cell activation. Methionine metabolic processes are closely linked to T cell differentiation and proliferation, with specific metabolic interventions significantly enhancing the development of stem cell‐like T cells for improved antitumor immunity. However, there is limited research on the mechanisms underlying the inhibition of methionine metabolism in stem cell‐like T cells. Direct competition for methionine between tumor cells and T cells results in SAM reduction and T cell dysfunction, making T cells sensitive to methionine as well. Conversely, inhibiting tumor methionine uptake normalizes methionine metabolism in T cells and restores their function [22].

In the context of our study, we hypothesize that the reprogramming of methionine metabolism in CD4^+^ T cells is associated with the maintenance of T cell stemness. Methionine restriction induces T cell dysfunction, while methionine restoration rescues T cell stemness. SAM, derived from the methionine cycle, is believed to mediate histone methylation and epigenetic remodeling during effector T cell differentiation [23]. H3K4me3 and H3K27me3 are implicated in the regulation of gene expression through a transcriptional pause‐release mechanism or by repressing transcription via the creation of a less accessible chromatin environment [24]. Although the underlying mechanisms require further investigation, methionine restriction reduced H3K4me3 marking at *Tcf7*, which altered CD4^+^ T cell stemness.

Mechanistically, Mapk13 phosphorylates Tcf1, which binds to the *Slc7a5* promoter to initiate its transcription. Slc7a5 imports methionine as a methyl donor, leading to induced H3K4me3 mark at *Tcf7* loci, thus maintaining T cell stemness. Our findings emphasize the role of the Mapk13‐Tcf1‐Slc7a5‐methionine metabolism axis in stem‐like CD4^+^ T cells. Additionally, these findings expand our understanding of metabolic reprogramming in stem‐like CD4^+^ T cells. Restricting methionine metabolism in stem‐like CD4^+^ T cells in chronic rejection grafts can be likened to removing the fuel from beneath boiling water. Inhibition of Mapk13 and MR diets may provide novel targets for enhancing long‐term graft survival.

## 4 Experimental Section

### 4.1 Patient Samples Collect

Specimens from healthy recipients and recipients with chronic graft rejection were obtained from the Organ Transplantation Institute of Tongji Hospital. This study was approved by the Ethics Committee of Tongji Hospital, Tongji Medical College, Huazhong University of Science and Technology (approval NO. 20231155). Informed written consent from all participants or next of kin was obtained prior to the research.

### 4.2 Single Cell Isolation and Enrichment

Renal tissues were minced in a digestion solution and incubated at 37°C with shaking at 250 rpm. Single‐cell RNA‐seq data were processed using the Seurat R package. Low‐quality cells were filtered based on standard quality control metrics, followed by normalization and identification of highly variable genes. Principal component analysis was performed, and the top principal components were used for graph‐based clustering and UMAP visualization. Cell types were annotated based on canonical marker genes and the CellMarker database. Differentially expressed genes among cell clusters were identified using established statistical methods.

### 4.3 Multiplex Immunohistochemistry Staining

For mIHC staining, 5 µm thick FFPE sections were stained using the 5‐color mIHC fluorescence kit (Recordbio Biological Technology, Shanghai, China) according to the manufacturer's protocol. The primary antibodies used were as follows: CD4 (cat# 48274, CST), CD8 (cat# 85336, CST), Tcf1 (cat# 2203s, CST), CD4 (cat# 25229, CST), CD8 (cat# 98941, CST), CD11b (cat# 93169, CST), and CD11c (cat# 97585, CST). DAPI was used for nuclear counterstaining. Stained slides were scanned using the Pannoramic MIDI (3D Histech, Hungary) to obtain multispectral images. CaseViewer was used to capture the images and identify all markers of interest.

### 4.4 Isolation of Splenocytes and T‐Cell Stimulation

Splenic CD4^+^ T cells were isolated using the Mouse CD4 T Cell Isolation Kit (cat# 480005, BioLegend) according to the manufacturer's protocol. To stimulate T cells, naive CD4^+^ T cells were seeded at a density of 2 × 10^5^ cells/well in 96‐well flat‐bottom tissue culture plates precoated with 5 µg/mL anti‐mouse CD3 antibodies (cat# BE0001, BioXcell). The culture medium was supplemented with 1 µg/mL anti‐mouse CD28 antibodies (cat# BE0015, BioXcell).

### 4.5 Immunoprecipitation and Immunoblot Assays

CD4^+^ T cells were isolated from spleen and stimulated with anti‐CD3/CD28 antibodies for 72 h. Cytoplasmic protein and nuclear protein separation were performed with Nuclear Protein Extraction Kit (cat# KTP3002, Abbkine) according to protocols. Subsequently, the protein lysate was incubated with primary antibodies or an IgG isotype antibody at 4°C for 12 h, followed by overnight incubation with Magna ChIP Protein A+G Magnetic Beads (Merck). The beads were washed and collected by magnetic separation and used for subsequent experiments. The primary antibodies for immunoprecipitation were: Mapk13 (cat# 271292, Santacruz), Tcf1 (cat# 2203s, cst), Phospho‐Threonine (cat# 9386, cst). The primary antibodies for immunoblot were: Tcf1 (cat# 2203s, cst), Mapk13 (cat# A7496, ABclonal), Slc7a5 (cat# A2833, ABclonal), Gapdh (cat# A19056, ABclonal), Mat2a (cat# A19272, ABclonal), H3K27me3 (cat# 9733, cst), H3K4me3 (cat# 9751, cst), H3 (cat# 4499, cst).

### 4.6 Mice and Surgical Procedures

Specific pathogen‐free C57BL/6J and BALB/c mice were obtained from Charles River Laboratories. Specific pathogen‐free Mapk13^fl/+^, Slc7a5^fl/+^ and CD4^Cre^ mice were obtained from GemPharmatech Co., Ltd. In vitro fertilization (IVF) breeding technology was used to generate Mapk13^−/−^ and Slc7a5^−/−^ mice. Mice were housed in a specific pathogen‐free facility at the Huazhong University of Science and Technology Research Institute in Wuhan, Hubei. All animal experiments conducted in this study were approved by the Huazhong University of Science and Technology Animal Care and Use Committee (approval NO. 202302004), in accordance with institutional guidelines for animal care and use.

The hearts of BALB/c mice were harvested and orthotopically transplanted into the peritoneal cavity of recipient mice. Recipient mice were treated with 10 mg/kg Ctla‐4 Ig (cat# BE0164, BioXcell) intraperitoneally 1 day before and 1 day after transplantation to establish a model of chronic coronary vasculopathy. In the heart graft survival experiment cohort, daily palpation was performed to monitor graft survival until the complete cessation of the heartbeat. In the pathological staining and mIHC experiment cohort, transplanted hearts were harvested at designated time points. In the flow cytometry experiment cohort, transplanted hearts, peripheral blood, and spleens were collected on Day 30 post‐transplantation.

The kidneys of BALB/c mice were harvested and transplanted into the peritoneal cavity of recipient mice. The transplanted kidneys were harvested on day 90 post‐transplantation.

### 4.7 Dual‐Luciferase Reporter Assays

The *Tcf7* sequence was cloned and inserted into the pCDNA3.1 plasmid. The 5'‐3' sequence, “ctgagggggcgggggcctttgcgagctgaaccaacagcagcggcgatgggcggagcctggagggcgggtaacggtagagataaaaggccgcgcgggcggg” was designed and inserted into the PGL3 plasmid. In black 96‐well plates, 293T cells were cultured to approximately 80% confluence, then co‐transfected with different plasmids for 12 h, and subsequently cultured for 72 h. Finally, the assay was performed using the Dual–Lumi Reporter Assay Kit (cat# RG088S, Beyotime). Firefly signals were normalized to Renilla luciferase to account for transfection efficiency.

### 4.8 Point Mutation and Lentiviral Infection

The pCDH‐Tcf1 plasmid was constructed for Tcf1 at T289. The threonine codon “ACA” was mutated to glutamic acid (E) “GAA” to mimic phosphorylation (pCDH‐Tcf1^T289E^) and to alanine (A) “GCA” to mimic dephosphorylation (pCDH‐Tcf1^T289A^). The pCDH plasmid was co‐transfected with psPAX and pMD2.G plasmids into 293T cells to package lentivirus, which was then used to infect CD4^+^ T cells activated for 24 h. CD4^+^ T cells were collected 48 h post‐infection, and RT‐qPCR was performed to detect the mRNA expression levels of *Tcf7* and *Slc7a5*.

The Tcf1 shRNA plasmid (cat# sc‐36617‐SH, Santacruz) or pCDH‐H1‐EF1‐Puro (scramble) plasmid was co‐transfected with psPAX and pMD2.G plasmids into 293T cells to package lentivirus, which was then used to infect CD4^+^ T cells activated for 24 h. CD4^+^ T cells were collected 48 h post‐infection, and RT‐qPCR was performed to detect the mRNA expression levels of *Tcf7* and *Slc7a5*.

### 4.9 Flow Cytometry

The antibodies used for flow cytometry were as follows: CellTrace Violet Cell Proliferation Kit (cat# c34557, Thermo Fisher), CD45‐PerCP‐Cy5.5 (cat# 564359, BD), CD11b‐BV421 (cat# 101236, Biolegend), CD4‐BV605 (cat# 563151, BD), CD8‐FITC (cat# 561966, BD), Tcf1‐AF647 (cat# 566693, BD), IFN‐γ‐PE (cat# 554412, BD), CD16/32 (cat# 101320, Biolegend). Transcription Factor Buffer Set (cat# 562574, BD) was used for membrane disruption and fixation. The Annexin V‐FITC/PI apoptosis kit (cat# AP101, Multi Sciences) was used for detecting cell apoptosis.

### 4.10 RNA‐seq, ChIP‐seq, and CUT&Tag‐seq

CD4^+^ T cells were isolated from the spleen and stimulated with anti‐CD3/CD28 antibodies for 72 h before being subjected to ChIP‐seq and RNA‐seq. The primary antibody for ChIP‐seq was Tcf1 (cat# 2203s, cst). Sequencing service was provided by Bioyi Biotechnology Co.Ltd. Wuhan, China. CUT&Tag‐seq was performed with the Hyperactive Universal CUT&Tag‐seq Assay Kit for Illumina Pro (cat# TD904, Vazyme) according to protocols. The primary antibodies for CUT&Tag‐seq were: H3K4me3 (cat# 9751, cst), H3K27me3 (cat# 9733, cst). Sequencing service was provided by Novogene Co.Ltd. Beijing, China. RNA‐seq, ChIP‐seq, and CUT&Tag‐seq data were processed using standard bioinformatic pipelines. For RNA‐seq, reads were quality‐filtered, aligned to the mouse reference genome, and quantified to identify differentially expressed genes. For ChIP‐seq, aligned reads were used to identify Tcf1‐binding regions using input controls. CUT&Tag‐seq data for H3K4me3 and H3K27me3 were processed to define enriched genomic regions. Integrative analyses were performed to relate transcriptional changes to Tcf1 occupancy and chromatin states.

### 4.11 Inhibitors

MAPK13‐inhibitors (cat# HY‐18850, MCE) were used in ex vivo experiments and the construction of the CAV model. FIDAS‐5 (cat# HY‐136144, MCE) and DZ2002 (cat# HY‐18620, MCE) were used in ex vivo experiments.

### 4.12 Statistics Analysis

Data are presented as mean ± SD. Sample sizes (n) are indicated in each figure legend. Comparisons between two groups were performed using a two‐tailed Student's *t*‐test, while multiple group comparisons were analyzed by one‐way ANOVA. A *P* value < 0.05 was considered statistically significant. All statistical analyses were performed using Prism version 9.0 (GraphPad Software).

## Conflicts of Interest

The authors declare no conflict of interest.

## Supporting information




**Supporting File**: advs73858‐sup‐0001‐SuppMat.docx

## Data Availability

Raw data files of RNA‐seq and ChIP‐seq experiments have been deposited in the NCBI Gene Expression Omnibus Database under accession numbers GSE247784 and GSE314157. Raw single‐cell RNA‐seq data have been deposited in the National Genomics Data Center under accession number PRJCA053651.
