# Supplementary material for: Targeting the Mapk13‐Tcf1‐Slc7a5 Axis via One‐Carbon Metabolic Regulation to Prevent Chronic Allograft Vasculopathy
Source: Adv Sci (Weinh). 2026 Jan 15;13(17):e20815. doi: 10.1002/advs.202520815 (PMC13042411; doi:10.1002/advs.202520815)
Supplement: Supplementary file 1 — Supporting File: advs73858‐sup‐0001‐SuppMat.docx [file ADVS-13-e20815-s001.docx]

Supporting Information

**Targeting the Mapk13-Tcf1-Slc7a5 Axis via One-Carbon Metabolic Regulation to Prevent Chronic Allograft Vasculopathy**

Wang Yi, Di Wu, Jing Liu, Shi Chen, Liu Song, Bin Xie, Aini Xie*, Peixiang Lan*, Zhishui Chen*

Supplemental table

**Table S1.** Patients’ metadata.

|  | Age | Gender | Weight (kg) | Creatinine (mg/dl) | Histopathological diagnosis |
| --- | --- | --- | --- | --- | --- |
| CCR1 | 58 | Male | 73 | 9.13 | TCMR |
| CCR2 | 43 | Female | 55 | 6.69 | TCMR |
| CCR3 | 55 | Male | 64 | 6.14 | TCMR |
| CCR4 | 49 | Male | 68 | 5.99 | TCMR |
| CCR5 | 37 | Male | 70 | 7.65 | TCMR |
| CCR6 | 40 | Female | 59 | 8.13 | TCMR |
| Non-rejection1 | 44 | Male | 65 | 0.77 | Non-rejection |
| Non-rejection2 | 28 | Female | 51 | 0.85 | Non-rejection |
| Non-rejection3 | 31 | Male | 55 | 0.63 | Non-rejection |
| Non-rejection4 | 39 | Female | 62 | 0.55 | Non-rejection |
| Non-rejection5 | 45 | Male | 70 | 0.68 | Non-rejection |
| Non-rejection6 | 36 | Male | 75 | 0.44 | Non-rejection |

Supplemental figures


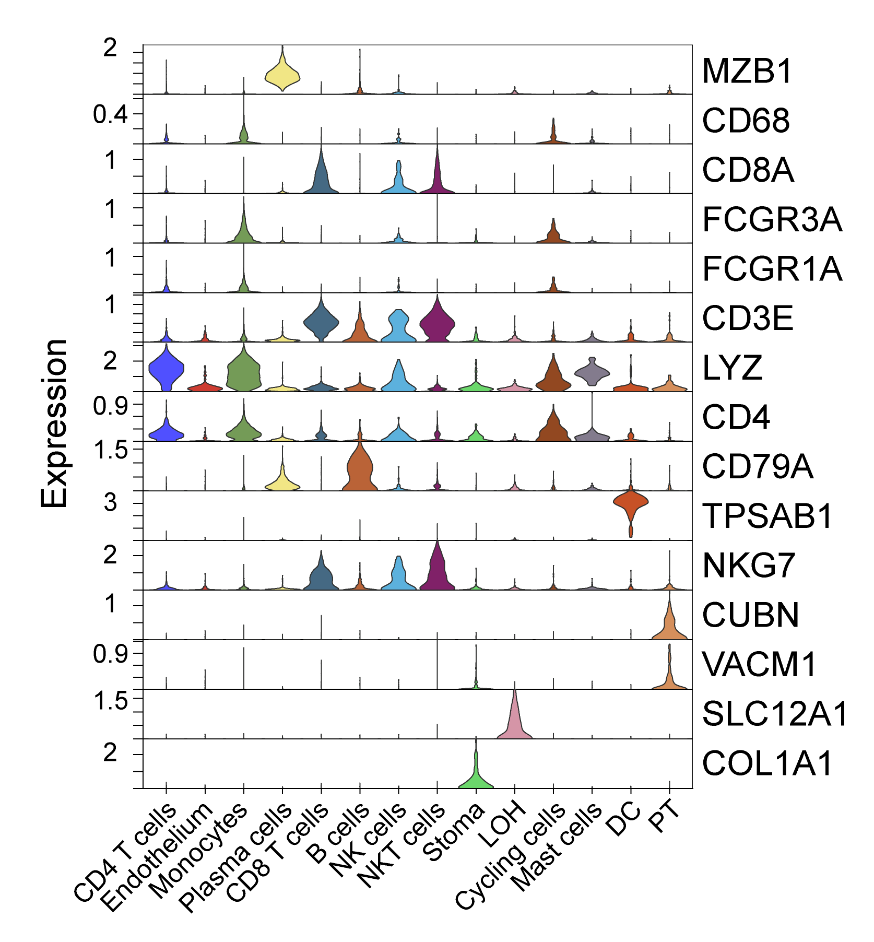


**Figure S1.** Violin plot highlighting lineage specific markers for each cluster identified in Figure 1A.


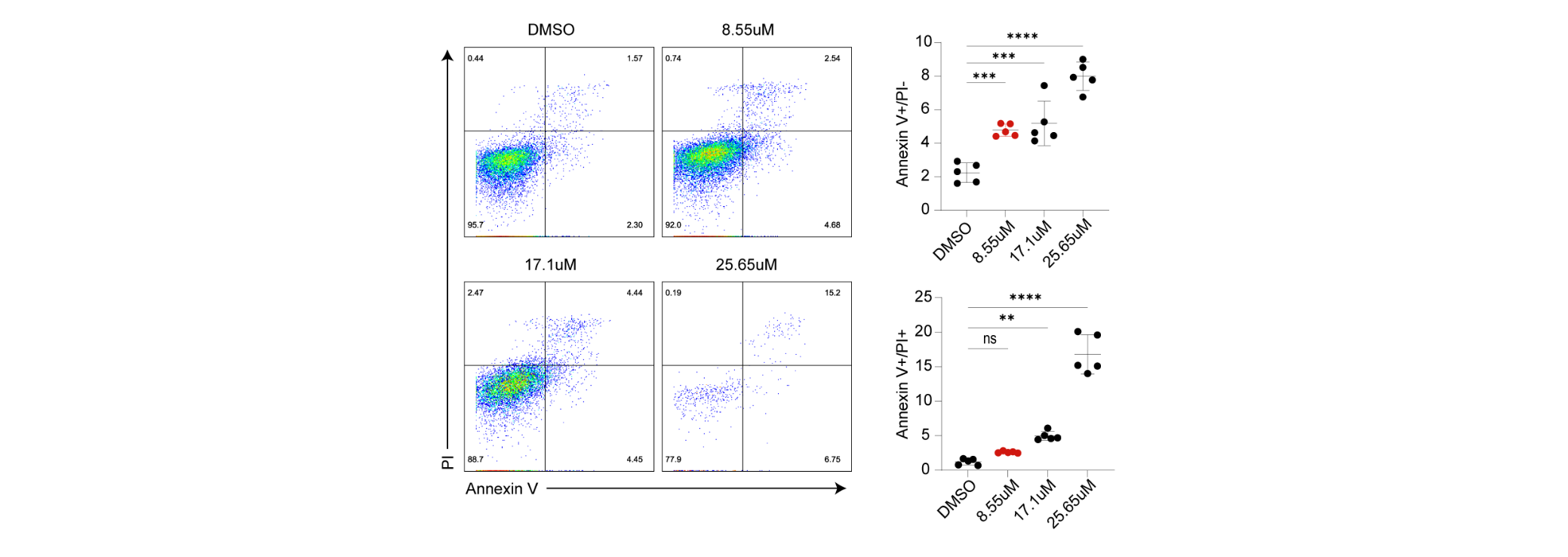


**Figure S2.** Apoptosis assay of CD4^+^ T cells treated with MAPK13-inhibitors at varying concentrations or DMSO was assessed by flow cytometry (n=5). ns, not significant, ***p*<0.001, ****p*<0.0001, *****p*<0.00001.


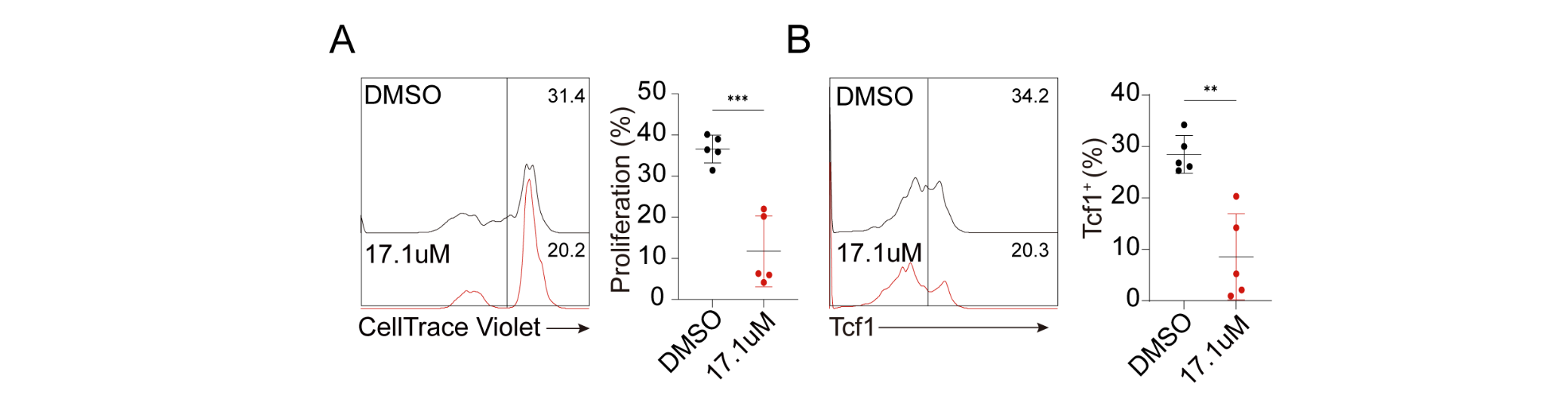


**Figure S3.** Splenic CD4^+^ T cells were isolated and used to perform mixed lymphocyte culture assays in the presence of MAPK13-inhibitors or DMSO to measure proliferation rates **(A)** and Tcf1 expression (B) (n=5). ***p*<0.001, ****p*<0.0001.


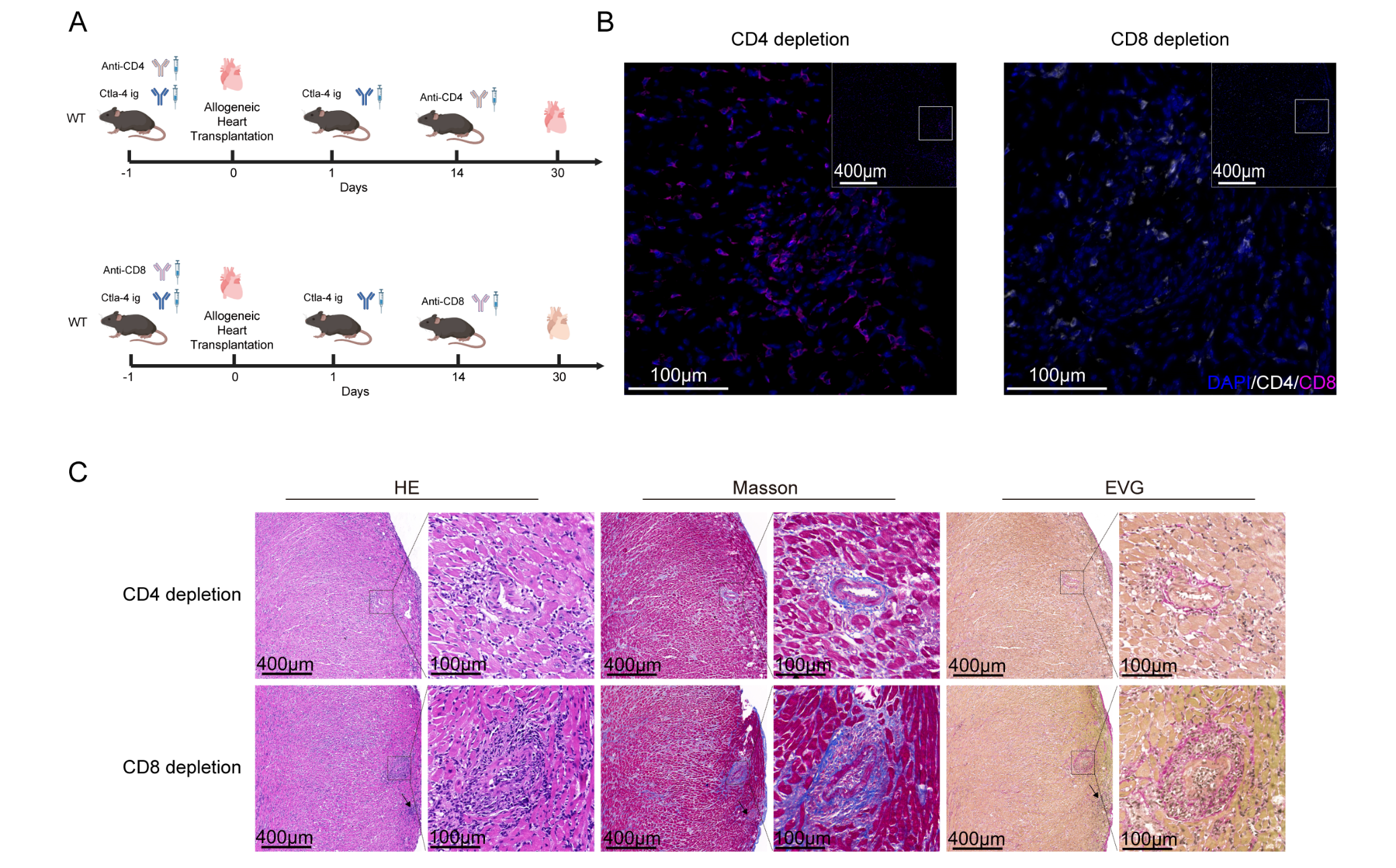


**Figure S4.** (A) Construction of the CAV model in CD4-depleted and CD8-depleted recipient mice. (B, C) Representative mIHC (B) and histological (C) images of transplanted hearts from CD4⁺- or CD8⁺-depleted recipient mice harvested on day 30 post-transplantation (n = 6).


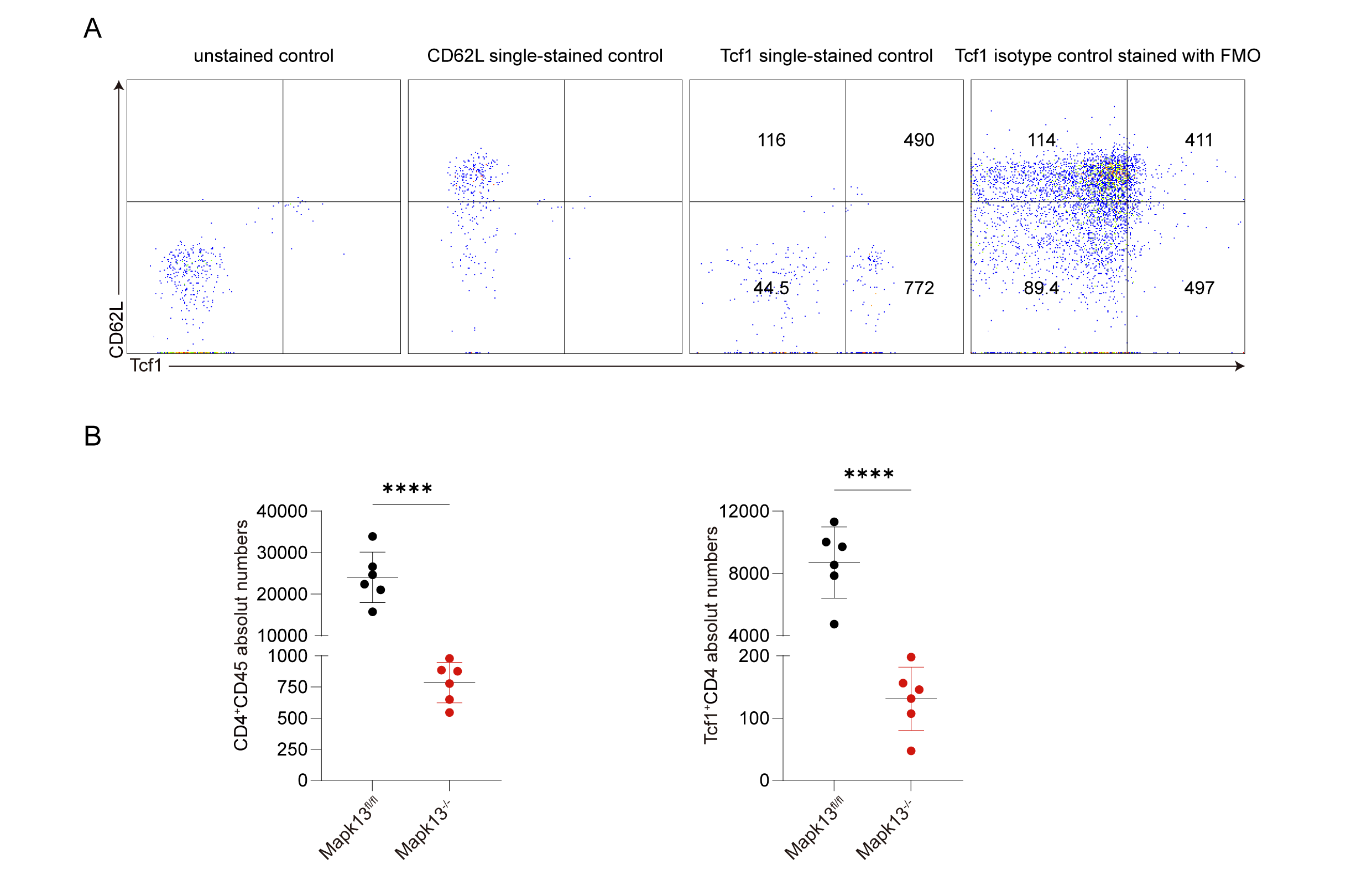


Figure S5. (A) Gating strategy and controls for the flow cytometry analysis of T cell subsets presented in Figure 3G-O. (B) Absolute numbers of infiltrating CD4⁺ T cells and Tcf1⁺CD4⁺ T cells in transplanted hearts of Mapk13^-/-^ recipient mice and Mapk13^fl/fl^ mice (n = 6).


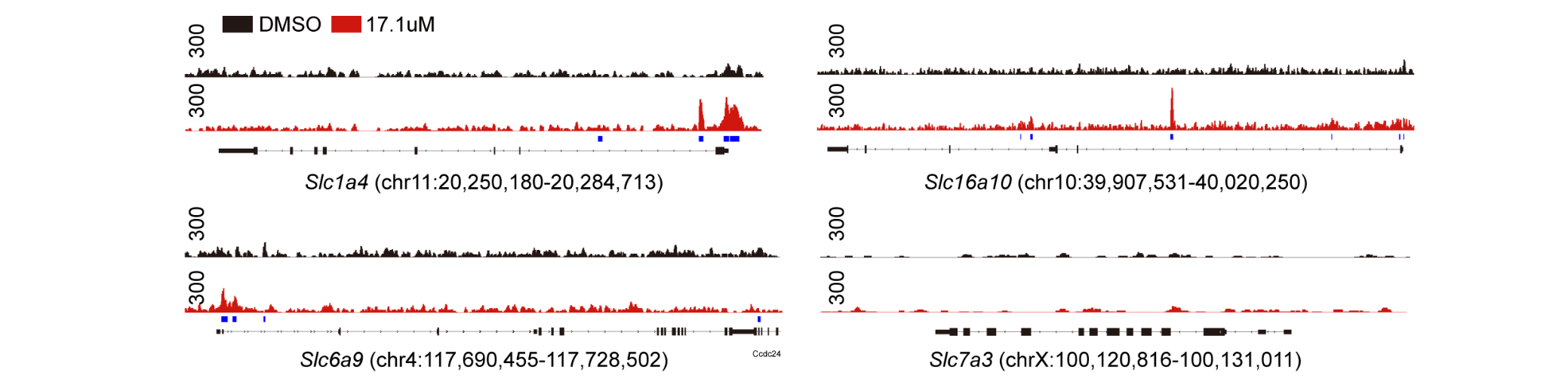


**Figure S6.** ChIP-seq tracks at *Slc1a4*, *Slc16a10*, *Slc6a9* and *Slc7a3* loci.


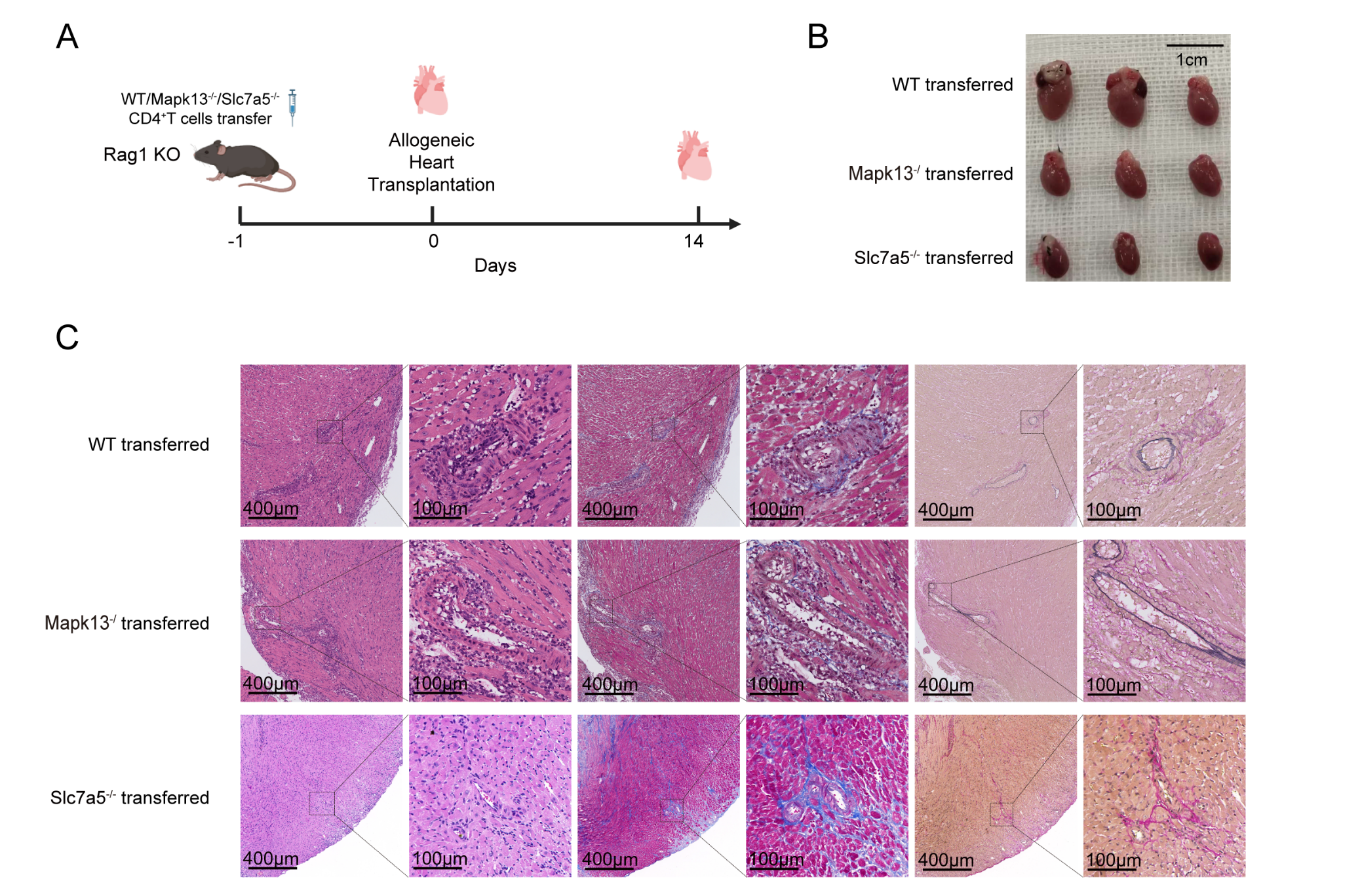


**Figure S7.** CD4⁺ T cells were isolated from spleens of wild-type, Mapk13^-/-^, or Slc7a5^-/-^ mice and adoptively transferred into Rag1 knockout recipients (3 × 10⁶ cells per mouse). Recipient mice subsequently underwent heterotopic abdominal heart transplantation using BALB/c donor hearts. (A) Construction of the CAV model established by adoptive transfer of CD4⁺ T cells. (B) Morphological comparisons of transplanted hearts harvested from Rag1^-/-^ recipient mice at day 14 after heart transplantation. (C) Representative histological staining of transplanted hearts (n = 6).


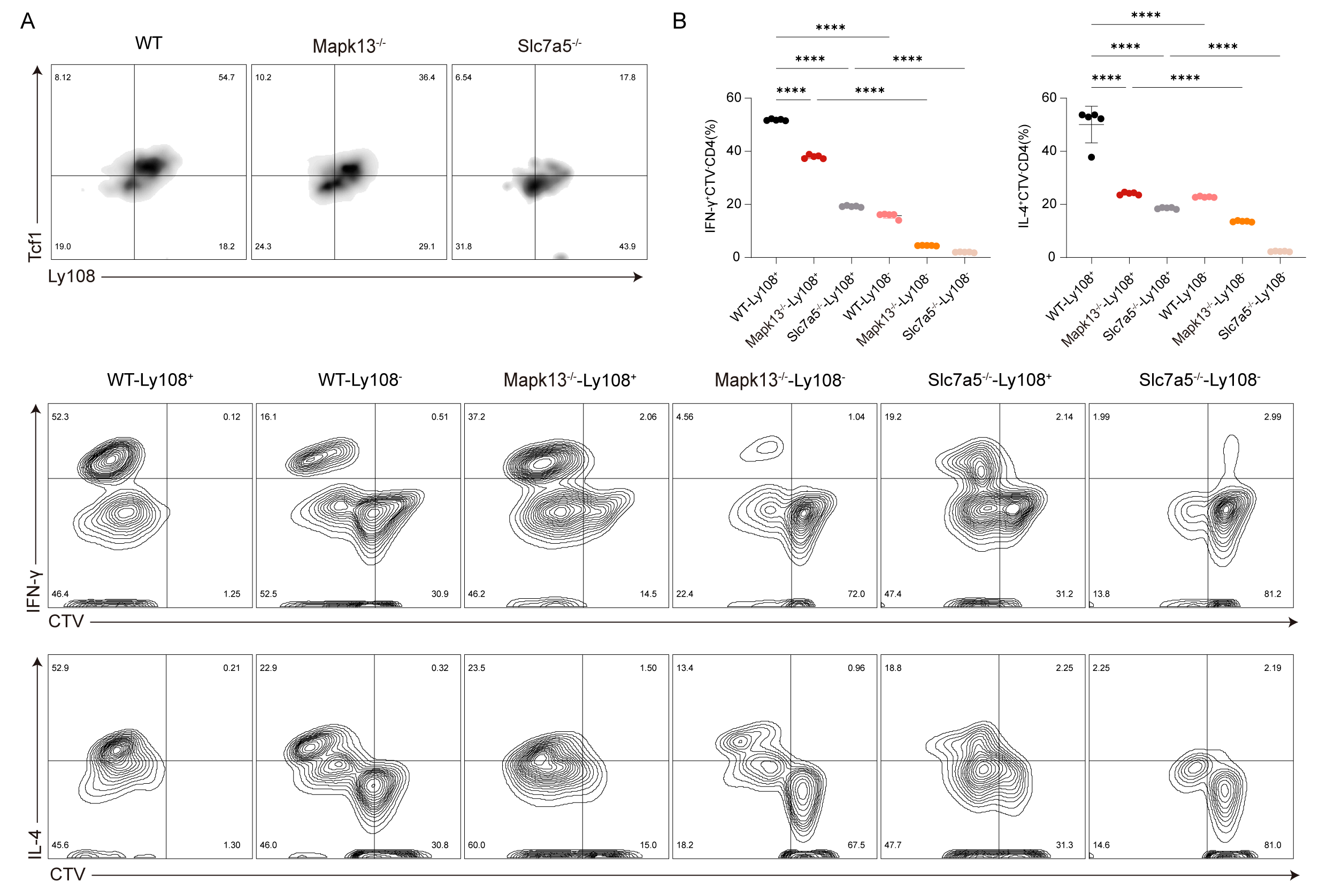


**Figure S8.** Splenic CD4⁺ T cells from WT, Mapk13^-/-^, and Slc7a5^-/-^ mice were stimulated with anti-CD3/CD28 for 72 h. (A) Representative flow cytometric analysis showing overlap between Ly108 and Tcf1 expression. (B) Ly108⁺ and Ly108⁻ CD4⁺ T cells were sorted, labeled with CTV, and cultured under Th1 or Th2 polarizing conditions for an additional 72 h. Representative flow cytometric analysis showing cell proliferation and differentiation. *****p*<0.00001.


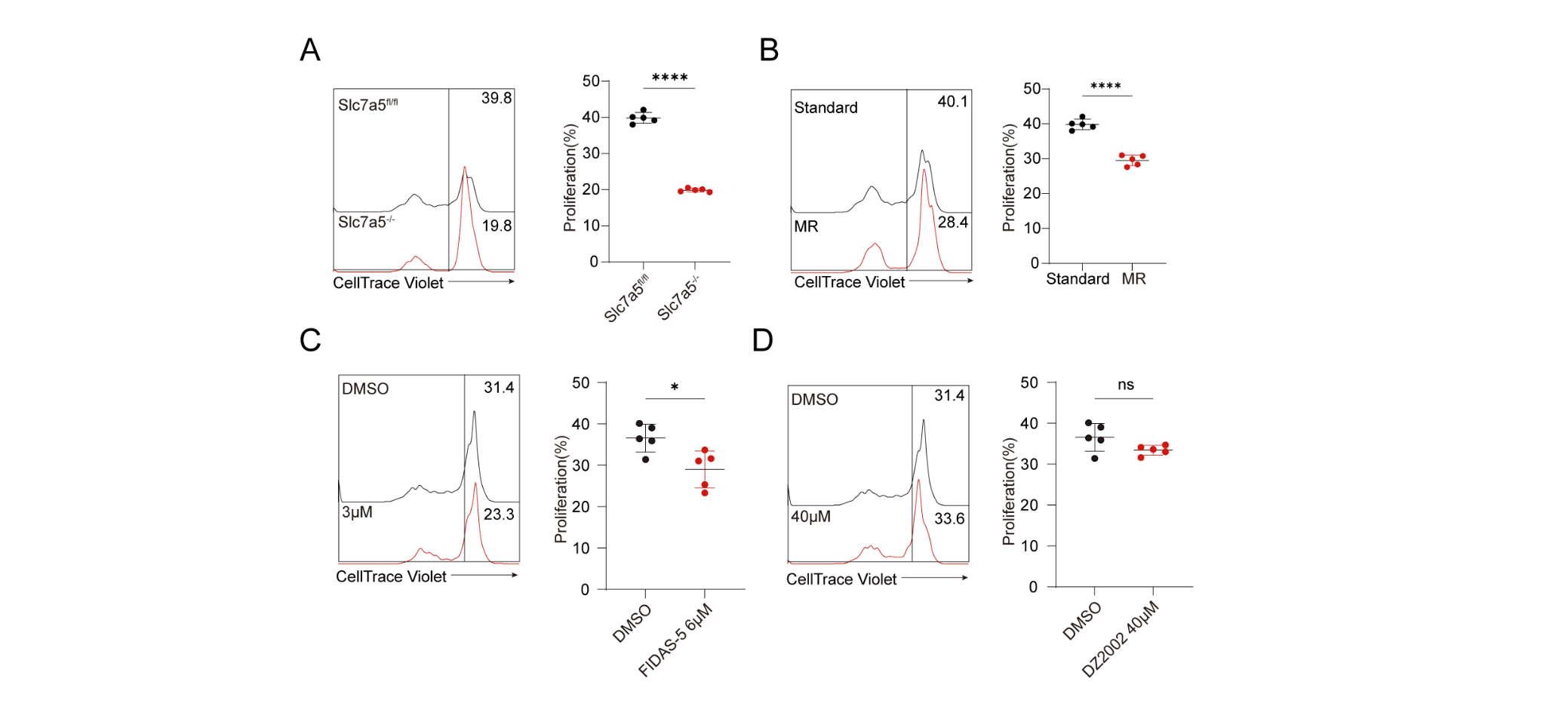


**Figure S9.** (A-D) CD4^+^ T cells were isolated from the spleen and used to perform mixed lymphocyte culture assays. (A) Cell proliferation of CD4^+^ T cells from Slc7a5^fl/fl^ and Slc7a5^-/-^ mice (n=5). (B) Cell proliferation of CD4^+^ T cells cultured in standard medium or MR medium (n=5). (C) Cell proliferation of CD4^+^ T cells cultured in the presence of FIDAS-5 or DMSO (n=5). (D) Cell proliferation of CD4^+^ T cells cultured in the presence of DZ2002 or DMSO (n=5). ns, not significant, **p*<0.01, *****p*<0.00001.


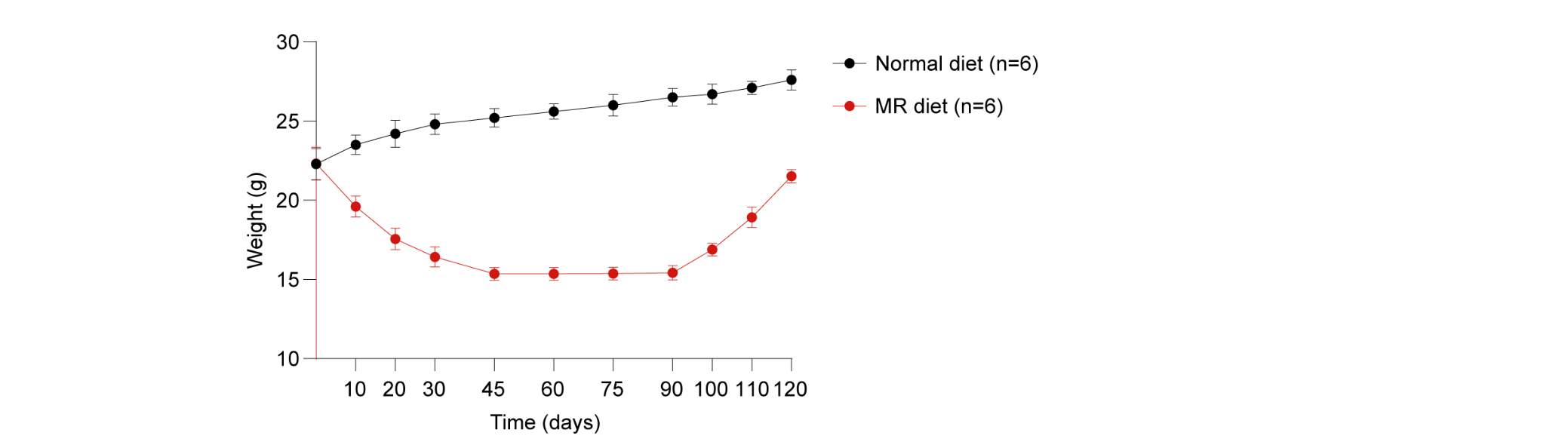


**Figure S10.** Body weight curves of recipient mice after heart transplantation.


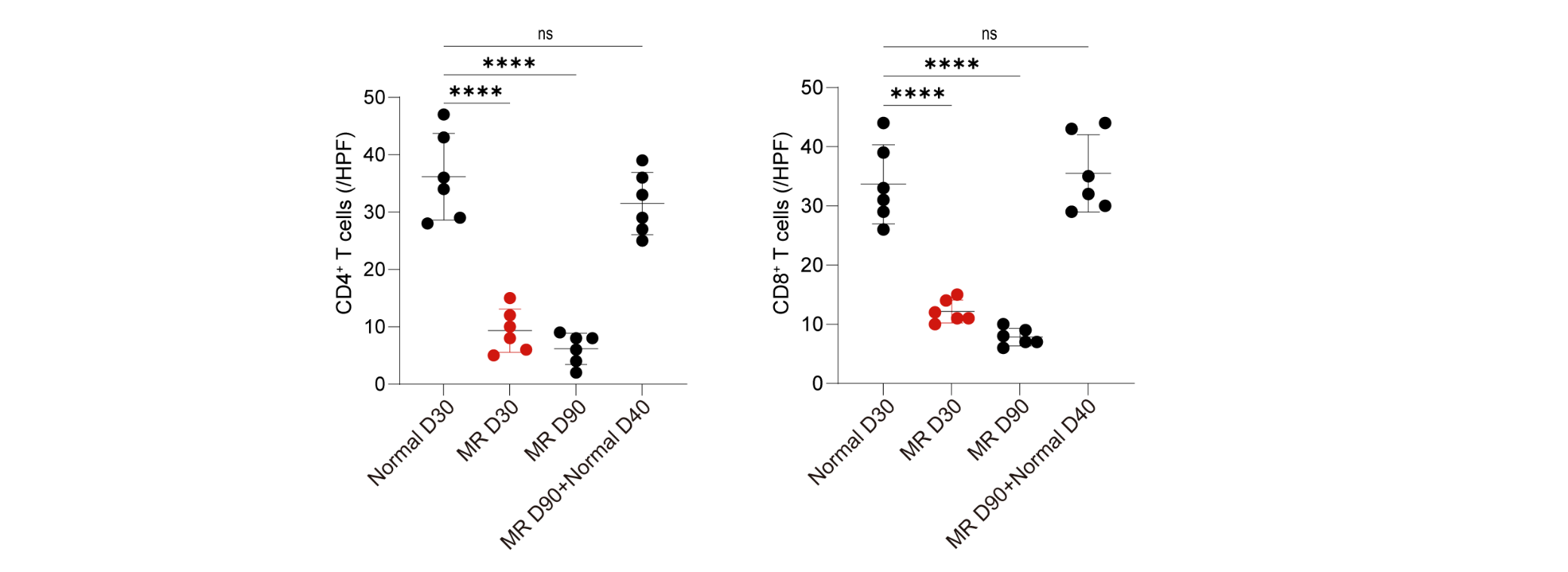
**Figure S11.** Proportions of CD4⁺ and CD8⁺ T cells shown in Figure 7F (n = 6). ns, not significant, *****p*<0.00001.


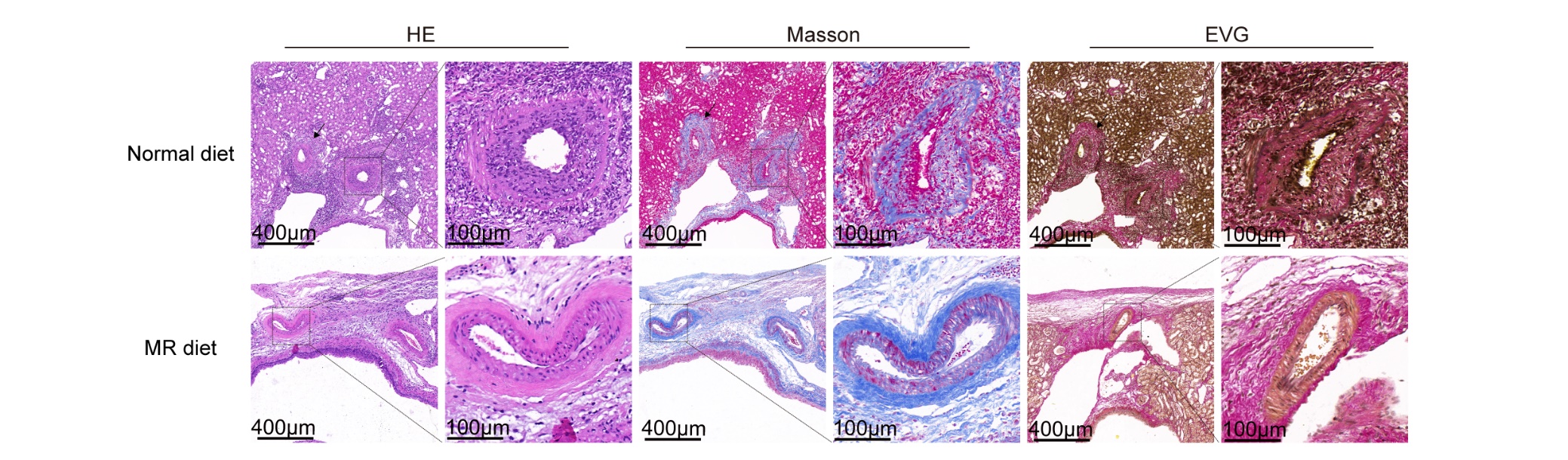


**Figure S12.** Representative histological stain of transplanted kidneys from normal diet and MR diet recipient mice harvested on day 90 post-kidney transplantation.

|  | Abbreviation |
| --- | --- |
| chronic allograft vasculopathy | CAV |
| chronic cellular rejection | CCR |
| multiplex immunohistochemistry | mIHC |
| methionine-restricted | MR |
| adenosylmethionine | SAM |
